# Reconfigurable Acoustofluidic Microvortices for Selective Microcargo Delivery

**DOI:** 10.1002/advs.202521612

**Published:** 2026-01-09

**Authors:** Lei Wang, Yiqiang Wu, Yiyang Tang, Hui Wei, Tianshi Lyu, Xin Zhang, Tian‐Yun Huang

**Affiliations:** ^1^ Laboratory for Micromachine Intelligence School of Advanced Manufacturing and Robotics PKU Research Center for Robotics Peking University Beijing China; ^2^ Department of interventional and vascular surgery Peking University First Hospital Beijing China; ^3^ Department of Sports Medicine Peking University Third Hospital Institute of Sports Medicine of Peking University Beijing China; ^4^ National Key Laboratory of Advanced Micro and Nano Manufacture Technology Beijing China

**Keywords:** intelligent micromachines, multi‐modal switching, reconfigurable microvortices, selective delivery

## Abstract

Microscale cargo delivery systems improve precise therapies by enabling delivery of microscale payloads deep into anatomical cavities through complex microvascular networks. Traditional methods face challenges, such as slow infusion, low payload throughput, and poor selectivity at microchannel bifurcations. Inspired by rotifers' adaptive predation, we introduce reconfigurable microvortex generators (r‐MVGs) featuring a frequency‐encoded acoustofluidic actuation mechanism, which can dynamically switch multiple modes under a single tunable ultrasound field. Diverging from conventional single‐mode microvortex or magnetic systems, the present approach leverages pairwise microbubble resonance encoding. This mechanism, wherein microbubbles of discrete sizes resonate at distinct acoustic frequencies, enables controlled attraction–repulsion transitions and programmable microvortex reconfiguration. These acoustically driven micromachines include a programmable active component and a passive nozzle connected by a rotary hinge controlled by paired microbubbles, creating directed microvortices. Simulations and experiments show that each bubble pair defines a unique frequency band, enabling multiple, independently tunable reconfigurable modes and real‐time switching between capture, release, and directional delivery. Using this platform, we successfully achieved selective payload guidance through a trifurcated microchannel network via active flow control. This frequency‐encoded r‐MVG system thus establishes a new paradigm for adaptive, multimodal, and high‐throughput acoustofluidic delivery in microfluidics, biomedicine, advanced manufacturing, and nano‐reservoir exploration.

## Graphical abstract

This work introduces a frequency‐encoded, reconfigurable acoustofluidic system that overcomes the low throughput and poor selectivity of conventional micro‐delivery methods. By exploiting discrete microbubble resonances to program adaptive microvortex modes, the platform enables real‐time switching and selective guidance of microscale payloads through complex channel networks, establishing a new strategy for precise, high‐throughput biomedical delivery.

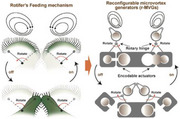

## 1 Introduction

**FIGURE 1:**
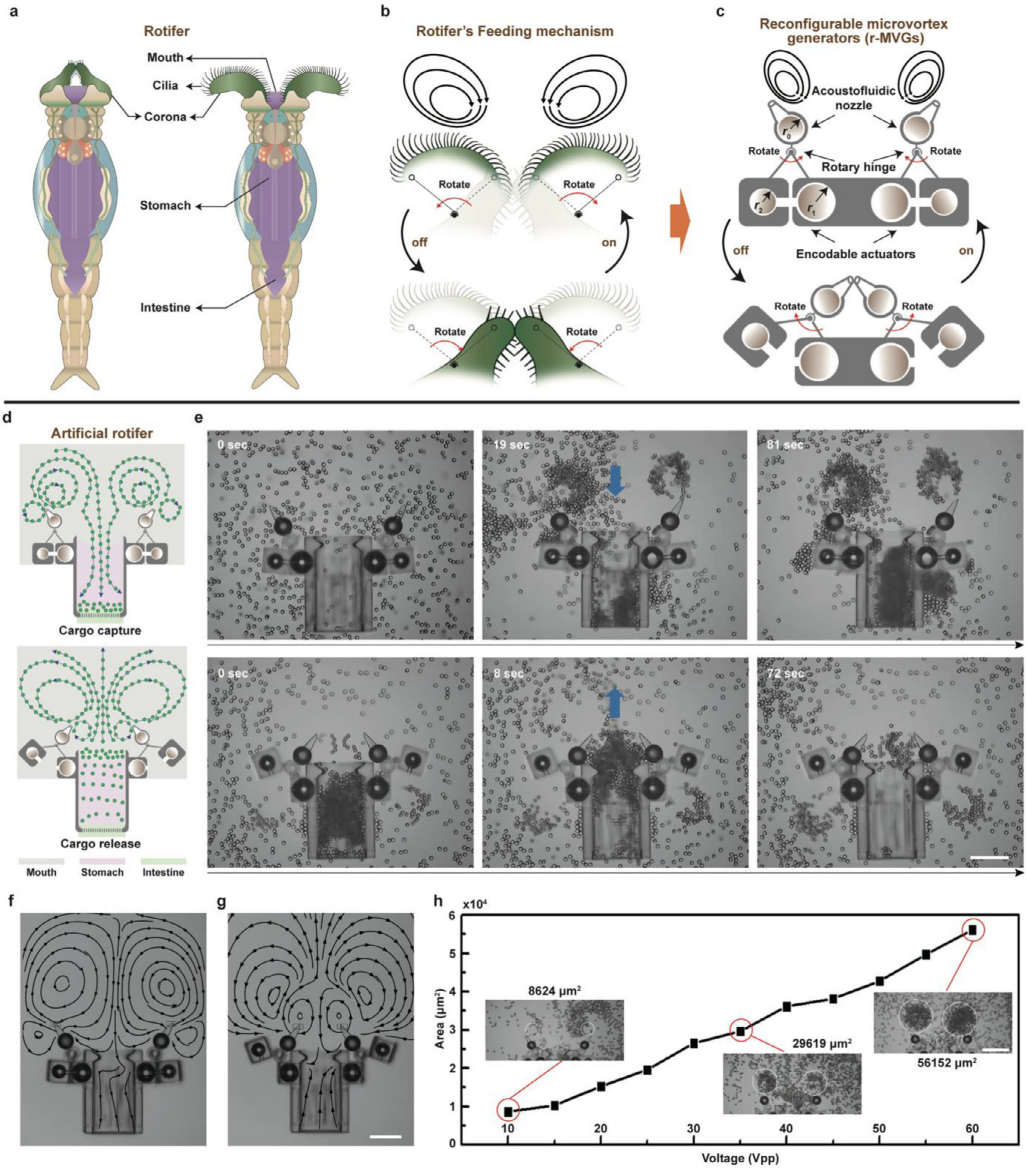
Rotifer‐inspired acoustically powered, reconfigurable microgenerator designs. (a) The rotifer's crown can alternate between open and closed configurations. (b) When open, the rotifer generates a complex flow pattern of counter‐rotating vortices through cilia at its mouth; this flow ceases when closed. (c) The microgenerator, inspired by rotifers, toggles acoustic microvortices on and off through mode switching. (d) Schematic of the compound micromachine's structure in capture and release modes. (e) Sequence of images showing cargoes captured at 119 kHz and released at 31 kHz. Scale bar: 100 µm. Numerical simulations of microstreaming patterns in the compound micromachine's (f) capture mode and (g) release mode. Scale bar: 100 µm. (h) Graphs depicting the area of captured cargoes (calculated using the area of the equivalent circle corresponding to the capture radius) versus excitation voltage, with insets highlighting the regions where cargoes gather at each voltage. Scale bar: 100 µm.

Rotifers are microscopic aquatic invertebrates, typically measuring approximately 100 microns in length, with a primary distribution in freshwater ecosystems. Over an evolutionary history spanning hundreds of millions of years, they have exhibited remarkable adaptability to diverse habitats, ranging from ephemeral puddles and flowing streams to stagnant lakes and even marine environments [1]. Central to their ecological success is a specialized feeding mechanism driven by cilia—minute, hair‐like organelles arrayed along the margins of their corona, a crown‐shaped structure surrounding the mouth (see Figure 1a). These cilia undergo coordinated, asymmetric beating patterns, which generate directional fluid flow under low Reynolds number conditions (characteristic of microscale environments). This flow dynamics results in the formation of a pair of chiral microfluidic vortices proximal to the oral region, enabling efficient entrainment and ingestion of organic detritus, bacteria, algae, and other suspended microorganisms [2].

Beyond their biological significance, the rotifer's cilia‐driven vortex generation has emerged as a paradigmatic model for bioinspired microengineering. Recent studies focused on recapitulating these microvortices artificially have yielded innovative applications in microscale technologies, including delivery, precise manipulation of microcargo (e.g., cells or nanoparticles), enhanced microfluidic mixing, and propulsion systems for microrobotics [3]. Notably, rotifers further demonstrate adaptive control over their feeding apparatus: they can reorient their corona in response to environmental stimuli (e.g., nutrient availability or predation risk), effectively opening or closing the mouthparts to activate or deactivate vortex formation (see Figure 1b). This ability to dynamically reconfigure structural function and switch between distinct microfluidic modes—from active feeding to quiescence—has garnered significant attention in bioinspired engineering. It has catalyzed the development of advanced artificial micromachines, such as reconfigurable lab‐on‐a‐chip devices and intelligent microrobots with adaptive functionalities, which are promising for diagnostics, screening, and lab‐on‐a‐chip [4, 5].

Utilizing microvortices for microfluidic delivery, pumping, and mixing—biomimetically inspired by the asymmetric ciliary beating of rotifers—represents a pivotal strategy in microfluidics research [6, 7, 8, 9, 10]. Contemporary approaches to generating artificial microvortices encompass the application of magnetic fields [11, 12], optical fields [13, 14], electrostatic fields [15], and integrated circuitry [10]. For instance, microvortices induced by millimeter‐scale magnetic soft cilia have been extensively investigated for fluid manipulation [16, 17]. However, precise directional control of such vortices using magnetic soft materials is inherently challenging, as it relies on preprogrammed magnetic moments and fixed ciliary architectures, limiting dynamic reconfiguration [18]. Light‐driven microvortices offer localized generation within confined microenvironments but suffer from practical limitations in complex systems: optical diffraction restricts spatial precision, while photothermal effects disrupt the synchronized hydrodynamic coordination of multiple vortices, impeding scalable operation [14]. Electrostatically activated microvortices enable localized pumping but require high operating voltages, rendering them incompatible with biomedical applications where biocompatibility and safety are paramount [15]. Furthermore, artificial ciliary platforms controlled by integrated circuits face constraints in environmental adaptability and multi‐channel scalability, primarily due to their reliance on photovoltaic power sources, which limit integration in resource‐constrained or opaque microfluidic systems [10]. These challenges underscore a critical unmet need in microfluidics: the development of scalable, reconfigurable, and individually addressable microvortices capable of operating within small, confined spaces.

Ultrasound fields are highly valued in microfluidics for their deep tissue penetration, versatility, cost‐effectiveness, biocompatibility, and capacity to generate substantial forces, thereby expanding their application scope [19]. Precise manipulation of acoustic pressure nodes enables effective control over particle ensembles within microchannels [20]. However, the spatial range and precision of this control remain constrained. The strategic introduction of sharp‐edge structures or microbubbles into microchannels can generate localized acoustic microvortices under ultrasound excitation [21]. These vortices, arising from intense hydrodynamic shear stresses associated with acoustic streaming, facilitate enhanced microcargo delivery [22], promote efficient fluid mixing [23], and assist in cell separation processes [24]. Despite these advances, most existing acoustic microvortex generators remain single‐state and lack real‐time configurability. They rely on static geometries or fixed resonant cavities, which cannot dynamically adjust flow direction or mode under a uniform excitation field [19]. Bioinspired ciliary bands exploit bidirectional acoustic microvortices for applications such as microrobotic navigation and cargo transport [25], although their fixed configurations inherently limit operational flexibility. A significant challenge lies in the current inability to achieve real‐time spatiotemporal control and dynamic switching of flow direction, which impedes the coordinated manipulation of multiple vortices and the realization of multimodal functionalities.

Here, inspired by the adaptive feeding mechanism of rotifers, we propose a new acoustofluidic strategy that encodes actuation (EA) frequency as a functional control variable. We introduce an ultrasound‐driven microvortex generator capable of frequency‐dependent reconfiguration, enabling programmable switching between multiple operational modes within a single acoustic field (see Figure 1c). This frequency‐encoded approach renders each microbubble pair an individually addressable unit, which resonates at a distinct frequency, thereby enabling decoupled control over attraction or repulsion forces. Building upon this core microgenerator, we further develop a compound micromachine system engineered for microcargo capture and on‐demand release, leveraging the vortices localized fluid manipulation capabilities. Beyond localized actuation, we present a scalable, acoustically augmented delivery platform designed to transport cargo over extended distances (millimeter to centimeter scale) and navigate through geometrically complex or occluded cavities, addressing a critical limitation of conventional microfluidic systems restricted to short‐range transport. This approach unifies long‐range transport, localized actuation, and multimodal programmability within a single, compact platform, thereby surpassing the limitations of previous single‐mode or magnetically encoded systems.

To enhance system versatility, we propose a novel actuation strategy utilizing ultrasound‐responsive, frequency‐encodable microbubbles, enabling individual microgenerators' frequency‐specific activation. This encoding mechanism allows for independent control and addressability of multiple microvortex arrays within a single system, overcoming cross‐talk challenges in multi‐unit actuation. By exploiting the interactive dynamics between two independently controlled vortex groups, we demonstrate the generation of four distinct microflow configurations, enabling multidirectional and selective fluid manipulation. These programmable flow modes are validated in a trifurcated microchannel network, where dynamic switching between flow directions is achieved, facilitating efficient cargo delivery to specific branch channels. Collectively, this work advances the field of acoustic microfluidics by integrating long‐range transport, localized actuation, and addressable control, with potential applications in lab‐on‐a‐chip diagnostics and microsurgery. Although the current work is primarily a proof‐of‐concept demonstration within microfluidic environments, its core elements of ultrasound‐driven microbubble actuation and two‐photon‐polymerized (TPP) microstructures are both grounded in well‐established biological foundations. Microbubbles have been widely used in vivo as contrast and therapeutic agents, while TPP‐fabricated microdevices have shown biocompatibility in tissue engineering. These precedents collectively support the long‐term translational potential of our acoustofluidic platform.

## 2 Results and Discussion

### 2.1 Rotifer‐Inspired Reconfigurable Microvortex Microgenerator

Inspired by the adaptive feeding mechanisms of rotifers within microscale habitats [1], we propose a reconfigurable acoustofluidic microvortex generator strategy. Emulating the rotational dynamics of the rotifer corona (Figure 1b), the generator comprises a compound micromechanism with a mechanically hinged, directional acoustofluidic nozzle. This nozzle passively rotates on a hinge joint, actuated by a pair of independently programmable rotary microactuators. Each microactuator integrates two encapsulated microspherical bubbles of distinct sizes, with the spherical bubble exhibiting greater stability. Under identical acoustic excitation, the phase difference between bubbles dictates their interaction: attraction occurs for phase differences below a quarter cycle, while repulsion dominates between a quarter and three‐quarters of a cycle.[37] Consequently, identical bubbles inherently attract, whereas dissimilar bubbles exhibit attraction or repulsion contingent upon excitation frequency. Innovatively, we extend conventional pairwise bubble interactions to programmable microbubble arrays via cross‐frequency combination. This enables independently addressable attraction and repulsion configurations. The resultant motion is constrained by the mechanical limits of the rotary units, permitting a maximum offset angle of 70°. The directional nozzle is rigidly affixed to its microspherical shell, which houses a dedicated microbubble for microvortex generation. Importantly, the microspherical shells share identical frames, ensuring bubble size is the sole variable parameter.

Coordinated activation and spatial arrangement of the paired microgenerators enable the creation of tailored flow fields, producing counter‐rotating microvortices. Notably, when actuated at specific frequencies to bring the nozzles into close proximity under continuous ultrasonic stimulation, these microvortices exhibit cessation, as demonstrated in Figure 1c (Two‐dimensional schematic diagram of the structure). Furthermore, by modulating the external acoustic field frequency, the generator achieves bidirectional and reciprocal switching of its shape‐morphing operational mode. Complementing the actuation strategy, we optimized the microsphere geometry to enhance gas retention, improve operational stability, and facilitate manufacturability.

In our design, the biological functionality is translated into an acoustically controlled mechanical analogy. Specifically, the two counter‐rotating microvortices generated at the front of the reconfigurable microvortex generators (r‐MVG) emulate the paired ciliary vortices formed by a rotifer's corona during feeding. The ON or OFF switching of these acoustic vortices through frequency modulation parallels the opening and closing of the rotifer's corona, which governs feeding activity. Functionally, both systems rely on synchronized dual‐vortex fields to capture and direct suspended particles—food in the biological case, and microcargo in our engineered system. While the rotifer's apparatus is living and adaptively regulated by neuromuscular control, our r‐MVG achieves a comparable level of reconfigurability through purely physical control of acoustic parameters. Therefore, the analogy operates at the functional and dynamical levels rather than the biochemical one, establishing a clear mechanistic correspondence between biological vortex feeding and engineered acoustofluidic transport.

r‐MVGs were fabricated on a glass substrate via two‐photon polymerization lithography. Their acoustofluidic response to varying external acoustic field frequencies was subsequently characterized. The substrate was positioned at the base of a fluidic chamber filled with an aqueous suspension containing 10 µm diameter fluorescent tracer particles. An acoustic field was generated by a piezoelectric transducer bonded to the opposing cover glass of the chamber; this transducer was driven by an external signal generator coupled through a voltage amplifier (see Figure S1). The propagation of the acoustic wave through the glass substrate into the fluid medium, and the resulting particle dynamics, were monitored and recorded using an upright optical microscope. Within the r‐MVG cavity, specifically at its narrowing opening, microbubbles were captured and stabilized by the solid‐liquid interface energy. When subjected to specific resonant frequencies of the external acoustic field, the air‐liquid interface of these confined microbubbles underwent oscillation, inducing strong microscale vortices in the fluid emanating from the cavity orifice. Crucially, the resonant response of each bubble pair defines a frequency‐dependent interaction mode (attraction or repulsion), thereby determining the mechanical configuration of the hinged nozzle and the resulting microvortex pattern. This enables reversible, frequency‐controlled morphological switching of the micromachine within the same continuous acoustic field, a feature absent in prior fixed‐geometry acoustofluidic systems.

To systematically investigate the acoustofluidic dynamics of the r‐MVGs, particle trajectories were tracked to characterize their response to microvortex motion induced around the nozzle pair. Experimental parameters included acoustic excitation frequencies spanning 10–200 kHz and peak‐to‐peak voltages ranging from 10 to 40 V, enabling the observation of frequency‐dependent vortex behavior. At approximately 31 kHz, the external acoustic field drives mutual attraction between microbubble pairs in the active region, concomitant with the opening of passive nozzles. This synergistic effect establishes a stable, time‐averaged vortex field between the nozzles. As visualized by dashed lines in Figure S2a, tracer microparticles reveal two counter‐rotating microvortices whose interaction generates a converging flow at their junction, where fluid velocities are enhanced as the flow is drawn inward. In contrast, increasing the frequency to 119 kHz triggers repulsion between active microbubble pairs, prompting the nozzles to rotate toward each other. This geometric reconfiguration markedly attenuates the reverse microvortex flow around the nozzles, ultimately leading to complete vortex dissipation (see Figure S2b).

Building upon the microvortex generator design prototype described earlier, we developed a bioinspired compound micromachine, termed an “artificial rotifer”, capable of precisely capturing and releasing microparticles. This micromachine comprises three principal functional components: a mouth, a stomach, and an intestine (Figure 1d). The mouth captures nearby microparticles, entraps them within a microfluidic vortex, and subsequently transports them into or out of the central cavity. The stomach serves as a cargo storage chamber, while a microporous mesh integrated within the intestine facilitates size‐based cargo filtration. To assess the efficacy of the rotifer design, we employed finite element analysis to simulate its microfluidic behavior (Figure S3). By modulating the amplitude of the applied acoustic excitation field, we controlled the deformation magnitude of the microbubbles, thereby simulating their characteristic oscillatory flow patterns. The simulations elucidated the formation of rotational microvortices and corresponding flow velocity distributions around the microgenerators during distinct operational modes (capture: Figure 1f; release: Figure 1g). Notably, these simulated flow patterns closely matched experimental observations, as evidenced by the strong agreement between simulation results and actual measurements presented in Figure 1e.

In capture mode, an external acoustic field operating at 31 kHz induces attractive forces, causing a pair of microbubble units within the active actuator to undergo coalescence. Simultaneously, two passive nozzles open, generating symmetrical microvortices. The interaction of these vortices at their interface produces a converging flow directed toward the stomach chamber. This flow regime adheres to low Reynolds number microfluidic principles (characteristic of microscale environments, ∼10^−3^), where viscous forces dominate, and inertial forces are negligible. Microparticles exhibit millisecond‐scale responsiveness to the initiation or cessation of ultrasonic excitation (Figure S4). When ultrasound is active, proximal microparticles are entrained into the microvortex flow field and captured. The effective capture radius is controllable by modulating the acoustic excitation voltage, as depicted in the flow profile (Figure 1e). While a single microvortex typically confines microparticles to a closed streamline (Figure S5), the interaction of the dual microvortices exerts an inward force on the outer streamline. At steady flow velocities, the consequent reduction in rotation radius increases the centrifugal force acting on the microparticles. This enhanced centrifugal force propels the cargo into the micromachine's designated storage area. Progressive accumulation of microcargoes within this storage region occurs over time (Movie S1). Integrated filter holes permit fluid drainage during filling until the storage capacity is reached, resulting in narrow interstitial gaps between densely packed cargoes. Theoretically, the stomach chamber's storage space can accommodate over 2800 microcargoes of 10 µm diameter.

We further investigated the capacity of artificial rotifers for anti‐flow regurgitation, applicable to tasks such as microcargo delivery and in vivo tissue biopsy. When subjected to an external acoustic excitation frequency of 119 kHz, microbubble pairs within the actuation unit undergo mutual repulsion. This interaction triggers a reconfiguration of the micromachine, initiating its microcargo release mode (Figure 1d). Crucially, within the passive units, the microvortices reverse their rotational direction compared to their initial motion during cargo capture. Streamline analysis during release revealed the following sequence: cargo proximal to the storage opening is initially entrained by the microvortex generated via the passive nozzles. Subsequently, the cargo adheres to the streamline flow until expulsion from the microgenerator occurs. As the influence of the microbubbles attenuates with increasing distance, cargo particles situated farther from the source progressively detach from the vortices and disperse into the surrounding medium (Movie S2). Within 72 s, the storage area was fully evacuated of cargo.

To evaluate the high‐throughput capture and release capabilities of the compound micromachine, we conducted systematic assessments of its cargo capture efficiency across a range of acoustic excitation voltages. Performance was quantified based on the spatial extent (volume) of cargo concentrated within the microvortices generated by the micromachine. An expanded cargo zone signifies stronger attraction forces, accelerated cargo transport, and increased cargo density, collectively enhancing capture efficacy. As demonstrated in Figure 1h, the cargo area increased approximately linearly with acoustic field strength. Increasing the voltage from 10 to 60 V resulted in a 551% expansion of the cargo area, demonstrating that capture efficiency can be effectively modulated by controlling the acoustic energy input.

### 2.2 Rotifer‐Inspired Microrobotic Delivery System

Microcargo delivery systems represent a pivotal advancement in interventional therapies, facilitating the precise transport of microscale payloads—including microdrugs, embolic microspheres, and microrobots—into deep anatomical cavities via minuscule, tortuous blood vessels. Unlike biological drug‐delivery processes, which involve transport, cellular uptake, and molecular release, our work focuses on the mechanical routing of cargos at the tens‐of‐microns scale in engineered microchannels. This concept is distinct from biological targeted therapy, as our system does not address intracellular uptake or biochemical release, but instead demonstrates high‐precision spatial routing analogous to interventional catheter‐based delivery. Such biomedical applications necessitate stringent spatiotemporal control over external actuation fields to ensure therapeutic efficacy and minimize off‐target effects [26, 27, 28]. At the microscopic vessel scale, fluid flow dynamics are governed by the Hagen–Poiseuille law [29], which dictates that the pressure required to propel fluids through microcatheters escalates precipitously with decreasing vessel diameter, scaling inversely with the fourth power of the diameter. This relationship renders conventional injection methods particularly challenging for micrometer‐scale capillaries, where even minor reductions in lumen size can lead to prohibitive pressure requirements [20, 30, 31, 32]. Despite their potential, current microrobotic systems face critical limitations that hinder clinical translation. Foremost among these is restricted payload capacity, which directly impacts the achievable therapeutic dosages and thus the efficacy of treatments. Their operational ranges are also constrained by rapid energy dissipation, limiting their ability to traverse large distances within the vasculature.

Recent innovations in active delivery mechanisms have sought to address these challenges, with notable progress in magnetic actuation. For instance, magnetic microtubular guidewires have been developed to transport magnetic cargo via surface‐mediated interactions, triggered by alternating magnetic fields [33]. While this approach demonstrates promise, it is inherently limited to magnetically responsive payloads, restricting its applicability to a narrow subset of therapeutic agents. Moreover, delivery efficiency is highly sensitive to the frequency and strength of the rotating magnetic field, requiring precise calibration to optimize cargo mobilization. A further drawback is the inherent tendency of magnetic cargoes to aggregate; as the number of cargo particles increases, interparticle magnetic interactions promote clumping, obstructing microvessels and reducing overall delivery efficiency by impeding individual particle mobility and targeting accuracy.

**FIGURE 2:**
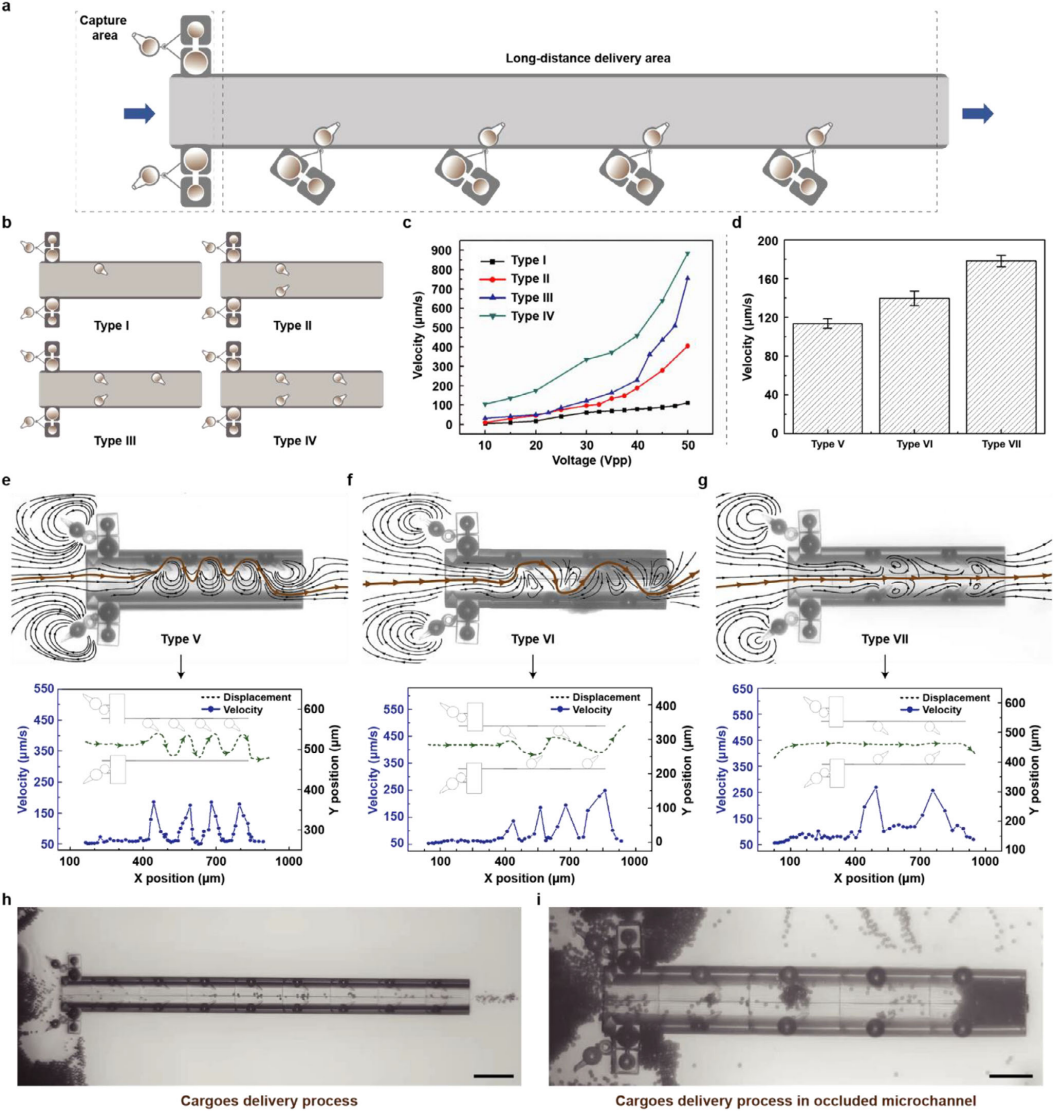
Rotifer‐inspired acoustic delivery. (a) Schematic of the long‐range acoustic delivery channel. (b) Diagram showing the delivery channel with varying amounts of microbubbles. (c) The correlation between delivery efficiency and ultrasound excitation voltage was tested with varying amounts of microbubbles. (d) The effect of microbubble arrangements on delivery efficiency at 20Vpp. (e) Delivery path and velocity of microbubbles under unilateral arrangement at 31 kHz and 20Vpp. (f) Delivery path and velocity under cross‐arrangement at the same frequency and voltage. (g) Delivery path and velocity under symmetrical arrangement at 31 kHz and 20Vpp. (h) Microscope images illustrating the long‐range delivery process, Scale bar: 200 µm. (i) Microscope images showing cargo delivery in an occluded channel, Scale bar: 100 µm.

As established, acoustofluidic reconfiguration of microstructures enables multifunctional microrobotic delivery systems capable of transporting diverse microscale cargo across substantial distances. These reconfigurable microvortex generators are typically arranged in confined, elongated microchannels (Figure 2a), forming vortex arrays that propel cargo via a fluidic relay mechanism. Furthermore, modulating excitation frequencies of the external acoustic field permits precise bidirectional control of cargo transport. Our investigation focuses on how microvortex positioning governs active acoustic delivery efficacy. A single generator nozzle produces paired chiral microvortices with opposing rotation. When inclined (Figure 2b), proximity to the microchannel wall constrains the adjacent vortex, reducing its radius, while the distal vortex expands. This symmetry‐breaking generates directional net flow through cooperative vortex interaction, facilitating cargo delivery. Increasing the number of generators enhances delivery speed, with minor velocity variations attributable to acoustic actuation voltage (Figure 2c). Notably, the Type IV configuration achieved 885 µm/s at 50 Vpp excitation—demonstrating the system's capacity for high‐throughput transport.

Experimental results establish that the spatial configuration of the microbubble generators determines the overall streamline distribution generated by cooperative microvortices. Even with identical microbubble quantities, different arrangements significantly impact cargo delivery efficiency, as illustrated in Figure 2d. Finite element simulations further elucidate the streamline patterns corresponding to distinct microvortex generator configurations—unilateral, staggered, or paired—shown in Figure 2e–g, respectively. The unilateral configuration produces sinusoidal delivery paths characterized by alternating acceleration and deceleration phases (Figure 2e). Conversely, the staggered configuration yields smoother, more streamlined paths (Figure 2f), while the paired arrangement results in a linear trajectory (Figure 2g). Comparative analysis confirms that shorter streamline lengths correlate with higher average delivery velocities (Figure 2d). And our system demonstrated excellent biocompatibility by achieving precise delivery of Drosophila cells with high viability (>95%) following ultrasonic actuation. These results confirm its safe operation and suitability for manipulating sensitive biological cargo (see Figure S18).

Within the rotifer‐inspired microrobot delivery system, adjacent microcargoes are initially entrapped by counter‐rotating microvortices generated at the inlet, facilitating their subsequent transport into the microchannel. Critically, an inclined nozzle integrated into the cavity wall, actuated by an external acoustic field, establishes a localized low‐pressure zone. This configuration enables microcargoes to overcome vortex confinement by exploiting hydrodynamic pathways (escape streamlines), thereby guiding them efficiently through the channel network toward ejection (Figure S6 and Movie S3). Notably, unlike pressure injection—whose efficacy diminishes over extended distances—this localized acoustic fluidic relay mechanism achieves high‐throughput microcargo transfer across significantly longer distances within confined, narrow channels (Figure 2h). Importantly, the system maintains full functionality even under channel blockage conditions (Figure 2i; Figure S7 and Movie S4). Furthermore, we will demonstrate how the rotifer micromachine's inherent capability for multimodal operation can be synergistically integrated with a programmable sequence of bubble activations to achieve selective delivery of microcargoes into designated microchannel branches.

### 2.3 Encodable Microvortex for Multimodal Acoustofluidic Reconfiguration

To clarify the multimodal behavior of the microvortex generator, we systematically investigate the microfluidic dynamics underlying interbubble interactions. According to the principles of microbubble acoustics, microbubbles driven by acoustic fields interact through the acoustic microflows they generate [34, 35, 36]. The dominant forces governing these interactions include a combination of acoustic radiation pressure from the background field and secondary effects arising from neighboring microbubbles acting as secondary acoustic sources. Notably, since the interbubble distance is significantly smaller than the wavelength of the acoustic signal, the primary acoustic radiation forces from the background field are deemed negligible in determining their relative movements [37]. The total force experienced by an active microbubble comprises three key components: forces resulting from microbubble interactions (*F_I_*), drag forces caused by acoustic streaming (*F_S_*), and secondary acoustic radiation forces (*F_R_*). To analyze these interactions, we model the microbubbles in an unbounded, incompressible liquid medium, assuming linear oscillations under small acoustic amplitudes while neglecting viscous and nonlinear effects. Under these simplifying assumptions, the total force acting on the microbubbles can be expressed as (Section S1) [37, 39–41]:

(1) $$F_{B}=F_{I}+F_{S}+F_{R}$$

In Equation (1), *F_I_* represents the acoustic radiation force due to the spatial gradient of acoustic pressure. *F_S_* represents the thrust from the flow—specifically, the self‐induced thrust caused by acoustic streaming from the volumetric oscillation of bubbles—and *F_R_* is the drag force acting on the microbubble, produced by the flow caused by neighboring microbubbles. In this study, the distance between the microbubbles remains consistently comparable to their size so that the bubbles are positioned close enough for their individual flow fields and pressure perturbations to interact. Microbubbles are significantly smaller than the sound wavelength, allowing the acoustic field to be considered uniform around each bubble because variations at that scale are negligible. Therefore, we assume that *F_R_* is independent of the distance between the microbubbles [38]. Given that acoustic streaming is a second‐order effect, its resulting thrust remains substantially smaller than the secondary acoustic radiation force at low drive levels with the microbubble approaching. Therefore, we have |*F_R_* | = |*F_S_* |. Based on the force balance equation [35, 36], the equation can be rewritten as:

(2) $$F_{B}\approx F_{I}=A\times G$$

(3) $$G=\frac{r_{1}r_{2}}{L^{2}}\frac{\left(1-\frac{\omega_{1}^{2}}{\omega^{2}}\right)\left(1-\frac{\omega_{2}^{2}}{\omega^{2}}\right)+\delta_{1}\delta_{2}}{\left[{\left(1-\frac{\omega_{1}^{2}}{\omega^{2}}\right)}^{2}+\delta_{1}^{2}\right]\left[{\left(1-\frac{\omega_{2}^{2}}{\omega^{2}}\right)}^{2}+\delta_{2}^{2}\right]}$$

where *G* > 0 indicates that the two microbubbles are in an attractive state.

**FIGURE 3:**
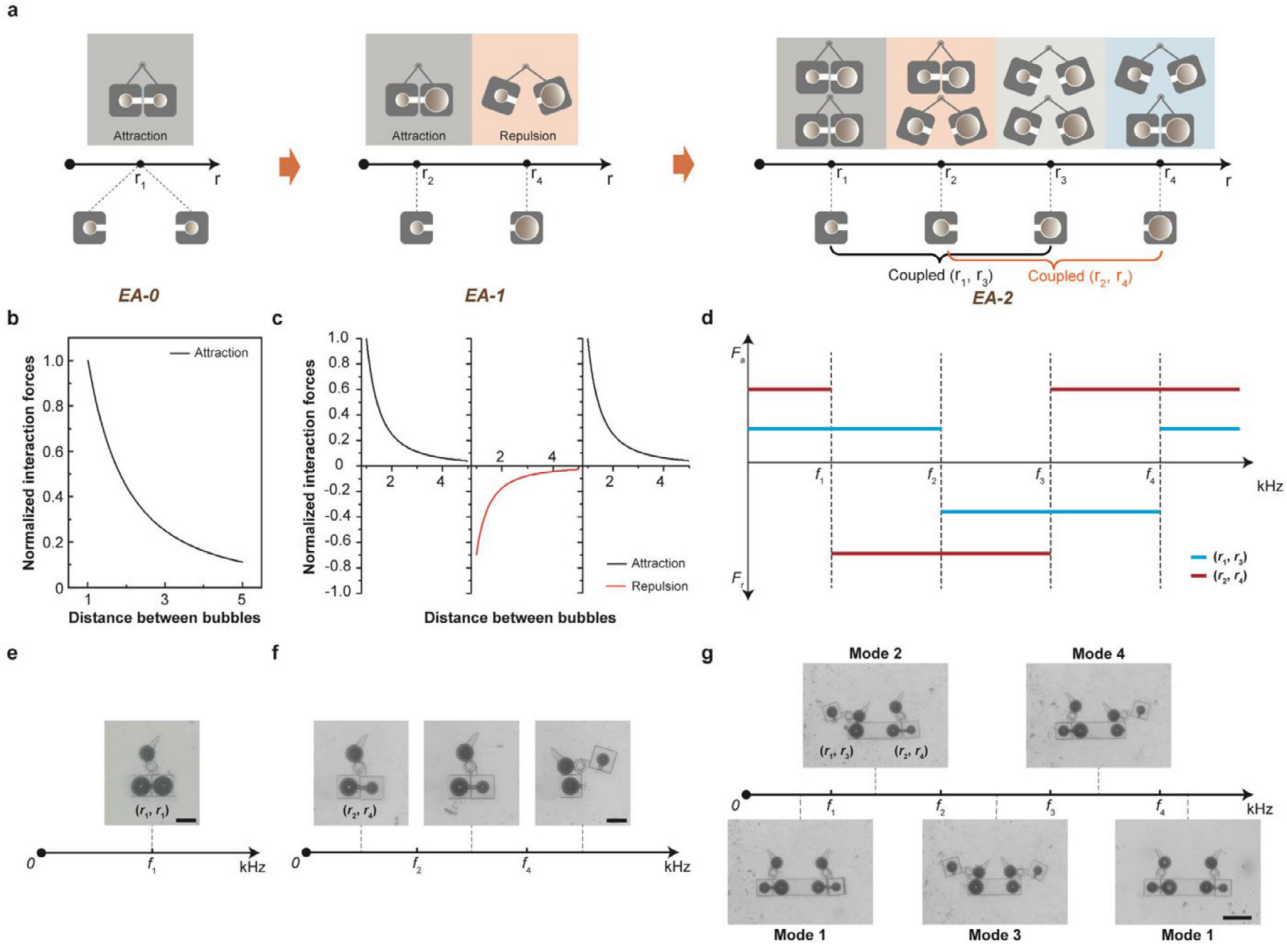
Principle of acoustic bandwidth division and experimental validation. (a) Design scheme for EA‐0, EA‐1, and EA‐2 within multi‐channel networks. (b) Numerical analysis of how the distance between identical microbubbles relates to the normalized interaction force. The black lines in the diagram represent the magnitude of the interaction force of the same microbubbles. (c) Numerical analysis of how the distance between different microbubbles relates to the normalized interaction force. The black and red lines in the diagram represent the magnitude of the interaction force for the different microbubble combinations. (d) A schematic illustrating the frequency‐dependent movement of microbubbles of varying sizes. The blue and orange curves indicate the distribution of the interaction force sign for two different microbubble pairings. On the positive semi‐axis of the y‐axis, phase differences lead to a repulsive force (Fr), whereas on the negative semi‐axis, the forces are attractive (Fa). (e) Microscopic images from an experiment showing the attraction behavior of EA‐0 within the ultrasonic frequency range. The selected frequencies (*f*_1_) are 109 kHz. Scale bar: 50 µm. (f) Microscopic images from an experiment showing repulsive and attractive behaviors of EA‐1 across an ultrasonic frequency range. The selected frequencies (*f*_2_, *f*_4_) are 125 and 181 kHz. Scale bar: 50 µm. (g) Microscopic images captured during an experiment where EA‐2 moved away from or attracted each other under different ultrasound excitation frequencies. The selected frequencies (*f*_1_, *f*_2_, *f*_3_, *f*_4_) are 109, 125, 148, and 181 kHz. Scale bar: 100 µm.

As shown in Figure S8, *r*_1_ and *r*_2_ are the radii of the microbubble pair, while *L* indicates the distance between them. ω is the acoustic excitation frequency, and ω_1_ and ω_1_ represent the natural frequencies of each microbubble, while δ_1_ and δ_2_ are the total damping constants for each bubble, respectively. *A* is a scalar related to the excitation amplitude that does not alter the force's direction. Equation (3) shows that identical microbubbles maintain in‐phase vibration, and the polarity of their interaction forces is consistent, regardless of the excitation frequency. However, interactions between microbubbles of different sizes are more intricate and depend on frequency. As depicted in Figure 3b,c, when the acoustic frequency is below their pair's natural frequency, dissimilar microbubbles oscillate in sync, resulting in mutual attraction. When the frequency falls between their two natural frequencies, they oscillate out of phase, leading to repulsion. If the frequency surpasses their natural frequency, they oscillate in phase again, causing attraction. In summary, identical microbubbles tend to attract each other, while microbubbles of different sizes can either attract or repel depending on the excitation frequency.

Numerical analysis establishes a direct correlation between the reconfigurable multimodality of microvortex generators and the actuation frequencies of microbubble pairs within an encodeable rotary microactuator. Introducing a size‐mismatched microbubble pair generates two additional operational modes. This frequency‐division encoding effectively transforms acoustic frequency into a digital addressable signal for actuation, allowing one r‐MVG unit to produce multiple discrete flow states. The natural frequencies of the pair act as spectral anchors, delineating distinct ultrasound frequency bands. By adjusting these bands, ultrasonic excitation enables independent control of attraction and repulsion forces. When microbubbles are identical, they oscillate in phase under ultrasound, resulting in consistent mutual attraction. Conversely, a size‐mismatched pair partitions the spectrum into three distinct intervals based on their natural frequencies, enabling two modal states—attraction and repulsion—as Figure 3a illustrates. Extending this principle, two different microbubble pairs can generate up to four distinct operational modes. Theoretically, n pairs partition the frequency spectrum into 2n + 1 intervals, thereby providing 2n control modes. This frequency‐based modal control significantly enhances microactuator manipulation. The scaling rule indicates that adding more pairs can indefinitely subdivide the frequency bands, contingent upon the ultrasonic system possessing sufficient resolution to excite the resonance of each individual microbubble.

To experimentally validate the principle of dividing frequency bands for multimodal reconfiguration, we developed three microbubble‐based microgenerators: r‐MVG‐0, a microvortex generator with a pair of identical microbubbles (EA‐0) with size of 30 µm (see Figure 3e); r‐MVG‐1, a microvortex generator with two different microbubbles (EA‐1) of sizes 18 µm and 26 µm (see Figure 3f; Figure S9); and r‐MVG‐2, which has four microbubbles (EA‐2) arranged in sequence at sizes 22, 30, 26, and 18 µm (see Figure 3g). As shown in Figure 3d, these setups generate distinct ultrasound spectral segments—one frequency band for EA‐0, three frequency bands for EA‐1, and five for EA‐2. During controlled acoustic stimulation (20–200 kHz, Vpp = 20 V), EA‐0 attracts continuously across the entire ultrasonic frequency band, and EA‐1 stayed attractively stable at 31 kHz and switched to repulsion at 119 kHz. For EA‐2, systematic frequency adjustments revealed four control states: continuous attraction at 31 kHz, left part repulsion at 119 kHz, right part repulsion at 137 kHz, and restored attraction in the left part at 171 kHz, as shown in Figure 3g and Movie S5. To further characterize the dynamic behavior and frequency response of the system, we conducted a continuous frequency sweep measurement (0–200 kHz) under fixed driving voltage. The resulting curve, provided in Figure S15, quantitatively shows the variation of vortex strength as a function of excitation frequency. Distinct resonance peaks correspond to the four operational frequencies used for modal switching. These peaks coincide with the admittance spectrum of the piezoelectric transducer, confirming consistent resonance coupling between acoustic input and bubble oscillation.

These experimentally observed transitions directly verify the theoretical encoding model and demonstrate frequency‐resolved, reconfigurable microvortex formation. By enabling multi‐band addressability without multiple transducers or field overlaps, this strategy provides a critical pathway toward scalable and programmable acoustofluidic manipulation. These observed transitions between attraction and repulsion states align closely with the predicted modal behaviors. Our findings demonstrate that dividing encoded frequency bands can be extended by increasing the number of differentially sized microbubbles, with each specific actuation pattern triggered by a unique ultrasound signal. Further, microbubbles of varying sizes exhibit precise frequency selectivity, detecting and responding to their resonant frequencies without observing modal interference during excitation. This reliable multimodal acoustic programmability enables the development of reconfigurable micromachine systems capable of executing multiple, distinct micromanipulation functions.

### 2.4 Selective Microcargo Delivery in Multi‐Branch Microchannels

Acoustic delivery has emerged as a leading non‐invasive and radiation‐free therapeutic modality, offering cost‐effective solutions for clinical applications [42, 43]. As a microscale active delivery technique, it exhibits significant potential to overcome the limitations imposed by the Hagen–Poiseuille equation, which traditionally restricts fluid flow dynamics in microconduits [44, 45, 46]. However, existing acoustic pumping technologies are confined mainly to unidirectional or bidirectional operation within single‐path conduits [47, 48]. In more complex delivery scenarios—such as microfluidic networks featuring branched channel architectures or multi‐compartment lab‐on‐a‐chip systems—unidirectional pumps lack reconfigurability and redirection capabilities, posing critical challenges for cargo transport [49, 50, 51, 52]. The selective delivery of microcargo to specific target branches within these intricate environments thus demands two key innovations: precise tunability of acoustic fields to spatially and temporally modulate force distributions, and robust adaptive control strategies that enable dynamic redirection of flow without reliance on mechanical valves. These advancements are essential to address the limitations of current systems and unlock the full potential of acoustic delivery in complex biomedical platforms.

**FIGURE 4:**
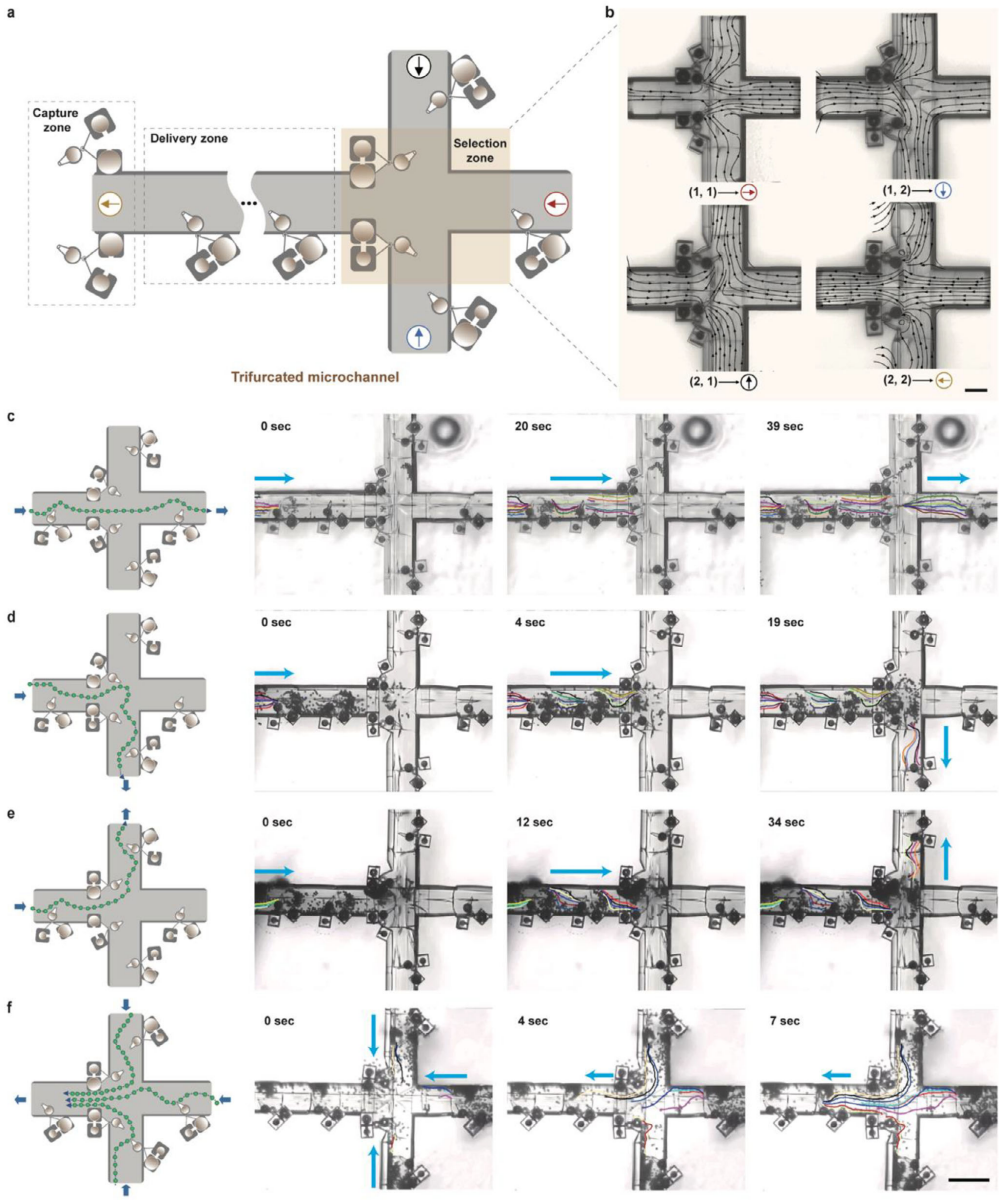
Selective acoustic delivery in a microchannel network. (a) Schematic diagram of the microchannel network, with arrows in different colors showing that the net flow direction in each channel can be independently controlled. The sizes of the active microbubble pairs in the capture, delivery, and selection areas are: (32, 30 µm), (30, 18 µm), and (30, 22, 26, 18 µm), respectively. (b) Numerical simulations of the microstreaming distribution of r‐MVG‐2 under various modes at the channel intersection. Scale bar: 100 µm. (d–g) With continuous excitation at 20 VPP, cargoes were selectively delivered to the target channels. The schematic on the left illustrates polystyrene cargoes (average diameter 10 µm) being delivered. The right microscope images display cargo aggregation and collective movement at different time points. Different colored lines in the channels track cargo trajectories, while the blue arrows outside the channels indicate cargo movement positions within the channels. Scale bar: 200 µm.

We employ an array of reconfigurable microvortex generators to construct an acoustically controlled, multi‐branched microchannel network within a single integrated microdevice. This network enables diverse microfluidic functions, including cargo capture, bidirectional transport, and branch‐selective delivery. By adjusting acoustic frequency, we selectively activate specific microbubble subunits within r‐MVG‐2, thus dynamically steering local flow directions and routing microcargo into designated branch channels. As illustrated in Figure 4a, the frequency‐sensitive design provides spatiotemporal control over cargo trajectories within the trifurcated network. The capture zone utilizes r‐MVG‐1 (Figure S8), operating in two distinct ultrasonic frequency modes to achieve efficient microcargo collection. Within the delivery zone, linearly arranged r‐MVG‐1 units facilitate reversible, bidirectional cargo transport through the microchannels. Finally, the selection zone functions as a hydrodynamic junction at the trifurcation, integrating r‐MVG‐2 for frequency‐dependent trajectory switching. This enables acoustic modulation to direct cargo into specific final delivery pathways.

We also demonstrate that highly precise, multimodal directional control of microfluidic manipulation can be achieved using a single, adjustable acoustic field—distinct from alternative methods that integrate multiple physical or chemical fields. To investigate how the microbubble group (r‐MVG‐2) configuration influences selective cargo delivery, we first analyzed flow distributions at trifurcated microchannel junctions under varying ultrasound frequencies. As illustrated by the directional arrows (black, orange, blue, and red) in Figure S10, each arrow denotes the net flow direction within its corresponding subchannel; the figure also depicts the r‐MVG‐2 setup and resultant flow patterns across different channels. Notably, microbubble arrangements play a pivotal role in guiding flow direction. This frequency‐programmed switching between channel outputs demonstrates selective cargo delivery in a multi‐branch microfluidic network achieved purely through acoustic frequency modulation, without physical valves or magnetic coupling. Specifically, symmetric microbubble pairs (2,2) and (1,1) generate symmetric microvortices at the intersection, which rotate in opposing directions to induce forward and backward net flows, respectively. In contrast, the asymmetric (2,1) pair triggers rapid fluid motion near microbubble apertures, where paired clockwise vortices collectively propel flow upward. Conversely, the (1,2) pair forms counterclockwise vortices, driving downward flow prior to r‐MVG‐2 activation. These observations suggest that the spatial placement of microbubbles within r‐MVG‐2 is the primary determinant of flow directionality. Our results highlight that strategic positioning of r‐MVG‐2 at channel intersections enables cargo delivery by directing flow into specific subchannels, thereby validating the potential of acoustic fields for precise microfluidic control.

We modeled flow patterns using a numerical perturbation method that simulates the fluid's response to acoustic excitation, combining first‐order harmonic and second‐order steady field components. The results, in Figure 4b, align with experimental observations and demonstrate flow directions at channel intersections consistent with our simulations, thus validating the method's effectiveness for evaluating fluid behavior across different r‐MVG‐2 configurations. Furthermore, we conducted experiments in 3D laser‐printed channels incorporating integrated r‐MVG‐1 and r‐MVG‐2 devices. For example, selective delivery tests in a triple‐branched channel confirmed precise transport (Figures S11 and S12, and Movie S6). The embedded microbubbles within the channels enhance device stability.

We further demonstrated that each of the four channels could be individually targeted. Experimental results confirmed that r‐MVG‐2, positioned at channel intersections, enabled precise steering of cargo flow upon acoustic activation. Without r‐MVG‐2, cargoes exhibited random movement into channels (Figure S13). When r‐MVG‐2 was activated, we systematically investigated cargo delivery across diverse configurations, focusing on intersection dynamics. In setups (2,2), (1,1), and (1,2), cargoes trapped within the capture zone were guided toward channel inlets by microvortices, subsequently entering the delivery area (Figure 4c–e; Movie S7). Upon reaching the first r‐MVG‐1 unit in the delivery zone, these cargoes underwent initial acceleration facilitated by divergent streamline escape regions, consistent with previously reported fluid dynamic mechanisms [53, 54, 55]. As additional cargoes entered the delivery zone, flow dynamics directed them from the first to the second r‐MVG‐1, driving secondary acceleration. Within the selection zone, cargoes were routed into specific channels based on r‐MVG‐2 activation, following distinct flow patterns (Figure 4c–e), thereby validating mode‐dependent channel selection.

Notably, in the (2,2) r‐MVG‐2 configuration, r‐MVG‐1 units in the delivery zone transitioned from repulsive to attractive, reversing flow direction within delivery channels. This reversal induced the draw of microcargoes from delivery branches into r‐MVG‐2 (Figure 4f; Movie S7), after which they traversed the delivery zone and exited through the trunk channel. These findings demonstrate that our reconfigurable microvortex system enables selective delivery of microcargo into target branches, representing positional control rather than biological selective delivery. Beyond experimental validation, complementary simulations further underscore the potential of untethered microfluidic control in multi‐channel fractal transport systems (Figure S14), highlighting the versatility of acoustic actuation for precise cargo manipulation in complex microfluidic architectures.

## 3 Conclusion

Inspired by rotifers' adaptive predation strategies, we introduce a novel design approach for reconfigurable microvortex generators capable of switching between distinct operational modes. These acoustic‐powered compound micromachines incorporate a programmable active actuator and a passive vortex‐generating nozzle, interconnected via a rotary hinge. The key innovation lies in the frequency‐encoded microbubble actuation mechanism, where bubble size determines resonant frequency and interaction polarity, enabling programmable, multimodal acoustic reconfiguration. Simulations and experiments verify that pairing microbubbles of differing sizes enables the precise generation of multiple independently adjustable and reconfigurable vortex modes. This mechanism allows a single global ultrasound field to orchestrate multiple addressable flow states (including capture, release, and selective delivery), demonstrating multimodal control unprecedented in current acoustofluidic systems.

Compared with magnetic encoding technologies [56], this acoustic approach offers greater simplicity, requiring only the identification of microbubble sizes exhibiting effective acoustic responsivity. Furthermore, multimodal reconfiguration is facilitated solely through a single tunable acoustic field. In contrast to magnetically controlled reconfigurable micromachines [57, 58], our fabrication process is also considerably simpler, involving only single‐step, single‐material 3D laser direct writing of the microstructures. A primary outstanding challenge lies in maintaining microbubble stability, as dissolution and concomitant size changes inevitably occur over time. While vapor deposition can markedly extend microbubble shelf life, an alternative strategy involves utilizing sharp‐edged microstructures with varying degrees of sharpness as actuators. Additionally, enhancing segmentation and actuation techniques for ultrasonic frequencies could expand the repertoire of achievable behavioral modes beyond those governed solely by microbubble size control. Although the current design space for microbubble actuators remains somewhat constrained, significant future opportunities exist, particularly in the realms of miniaturization and the optimization of spatial topology.

Selective microcargo delivery within a multichannel network underscores the system's capability for multimodal microfluidic manipulation. These results validate acoustically controlled microscale cargo transport as a promising strategy for high‐precision microfluidic manipulation, with potential relevance to catheter‐assisted localization of therapeutic materials rather than direct biological targeted therapy. This form of selectivity is positional rather than biological, and future integration with molecular recognition elements would be required to achieve biological targeting. In this work, the selectivity arises purely from engineering mechanisms and does not involve biological recognition such as ligand–receptor interactions or targeted cellular uptake. While the feasibility of this technique has thus far been demonstrated exclusively in ultrasound‐based microfluidic environments, its potential for broader technological translation is significant. Microbubbles and TPP‐fabricated microstructures have already been applied in biological or near‐in vivo contexts, suggesting that our approach can be extended toward biomedical use with proper material and ethical preparation. This promise stems from enhanced precision in microbubble encoding, which opens avenues for applications in reconfigurable intelligent micromachined systems, adaptive metasurfaces, and multimodal ultrasound sensing, among other emerging fields.

For future applications, the spatial configuration of the device can be flexibly adapted according to the target environment: (i) in vascular‐inspired or microcatheter systems, the generator may be embedded within the catheter lumen and deployed in blood vessels to locally reshape flow for precision delivery; (ii) in open or droplet‐based environments, it can be externally mounted on the chamber wall for flow manipulation and particle collection; and (iii) in microfluidic chips, it can be integrated directly into the channel walls as part of the microfabricated network.

## 4 Experimental Methods

### 4.1 Fabrication Methods

All microcargo delivery microdevices were fabricated using an advanced 3D laser nanolithography system (Nanoscribe GmbH). To achieve high‐resolution printing of intricate features, a 63× oil‐immersion objective lens (Zeiss, model 63×/1.4 oil DIC M27) was employed. This configuration enabled precise manipulation of the IP‐Dip photoresist (Nanoscribe GmbH). During the design phase, detailed 3D models of all microdevices were meticulously created using AutoCAD software. These models were subsequently processed using Nanoscribe's Describe software to generate instructions compatible with the nanolithography system. Printing parameters were optimized for structural fidelity: vertical slicing was set at 0.4 µm and horizontal slicing at 0.3 µm. A constant scan speed of 0.01 m/s and a laser power of 45 mW were maintained throughout the printing process to ensure effective photoresist polymerization. Following fabrication, unpolymerized residual photoresist was removed via sequential solvent immersion. The device was first developed in SU‐8 developer (PGMEA, propylene glycol monomethyl ether acetate, Sigma–Aldrich) for 15 min to dissolve unexposed resist, followed by a 10‐min rinse in isopropyl alcohol to yield a clean, well‐defined final microstructure.

The entire microgenerator was fabricated monolithically using TPP lithography without manual assembly. During design, micro‐clearances were introduced at the hinge interface to form a freely movable joint after photoresist development. Following fabrication, a microprobe gently released the movable part from the substrate under an optical microscope, breaking the temporary anchoring and allowing the nozzle to rotate. No post‐assembly of discrete parts was required. The maximum rotation angle permitted by the hinge constraint was approximately 70°, as illustrated in the exploded‐view schematic (see Figure S16).

### 4.2 Acoustic Field Generating System

The acoustic microsystem was fabricated on a glass slide (2.2 mm × 2.2 mm × 0.15 mm) and permanently bonded using a high‐strength epoxy resin (Devcon, 5‐min two‐component epoxy). Following fabrication, microchannels and microstructures underwent rigorous cleaning with isopropyl alcohol (IPA, Aladdin brand) to ensure optimal functionality and particulate‐free surfaces. The prepared device was then mounted on a microscope stage for analysis. A precisely calibrated mixture of deionized water and 10.0 µm tracer particles (10:1 v/v ratio) (polystyrene microspheres, Sigma–Aldrich) was introduced onto the microstructure to establish an acoustically responsive medium. Tunable acoustic fields were generated within the fluidic environment via a piezoelectric transducer (Murata, 7BB‐27‐4L0) driven by a signal generator (GW Instek, AFG‐2225 arbitrary function generator) and amplifier (Aigtek, ATA‐2041 high‐voltage amplifier) with a sinusoidal signal. This configuration enabled dynamic manipulation of acoustic wave properties. The entire assembly was integrated with an inverted microscope for real‐time visualization, facilitating high‐resolution observation and analysis of microscale interactions within the microfluidic domain.

To evaluate the stability of the microbubble, we characterized its lifetime. In deionized water, the microbubble maintained its volume for 28 min without excitation, while its operational duration under 25 V ultrasonic excitation was 12 min. To enhance stability, we employed PBS buffer, which significantly extended the maintenance time to 76 min without excitation and the operational duration to 33 min under the same excitation, representing an increase of over 170%. Moreover, the lifetime of microbubbles was correlated with the driving frequency. When driven near their resonance frequency, the oscillation amplitude reaches its maximum, significantly enhancing the rectified diffusion effect, which can lead to rapid bubble growth and thereby shorten the lifetime of bubbles within a specific size distribution. Conversely, when driven away from the resonance frequency, both the oscillation amplitude and the rectified diffusive mass flux decrease, consequently improving bubble stability and extending its lifetime.

Bubble sizing was carried out by isolating the in‐focus bubble silhouette from bright‐field and high‐contrast frames, followed by sub‐pixel edge detection and circular fitting in ImageJ to determine the instantaneous radius. Optical sizing was selected because it provides a direct geometric measurement that can be readily calibrated against micrometer standards and was widely used in single‐bubble characterization. Each bubble radius was measured independently three times, and the mean value was used in subsequent analysis to further reduce uncertainty.

The system's frequency tolerance was defined as the −3 dB (half‐power) bandwidth of the piezoelectric transducer. This provides a direct and measurable criterion for determining whether frequency drift can affect acoustic encoding. Bubble resonance selectivity was assessed by comparing this bandwidth with the natural frequencies of individual bubbles and with any interaction‐induced shifts.

### 4.3 Numerical Simulations

This study performed numerical simulations using COMSOL Multiphysics, a leading finite element analysis software. 2D models were constructed using triangular elements to ensure computational efficiency. For 3D geometries, tetrahedral elements were employed to simplify the meshing process while accurately representing complex structures. The acoustic field was modeled as linear first‐order fields derived from the Navier‐Stokes equations governing fluid motion. A second‐order perturbation term was incorporated to simulate acoustic streaming effects accurately and capture the resulting time‐averaged flow patterns. Pressure and velocity fields were computed using P1‐P2 composite elements: P1 elements utilized first‐order Lagrangian polynomials on triangles, while P2 elements employed second‐order polynomials, enhancing solution accuracy. These equations were solved using direct solvers, yielding stable and reliable results for both the first‐ and second‐order formulations. The research focused on microbubble‐induced acoustic streaming under static conditions, enabling a detailed analysis of the underlying hydrodynamic interactions. This approach provides fundamental insights into the influence of acoustic fields on fluid dynamics in the presence of microbubbles. We compiled statistics on all parameter material properties used in the COMSOL simulation, and a snapshot of the meshed computational domain, as shown in Figure S17.

### 4.4 Characterization of the r‐MVG Motion and the Generated Fluidic Flow

Experimental observation employed an optical microscope (Hirox RH‐2000, Japan) equipped with a high‐resolution CMOS camera. Video recordings obtained during experiments underwent comprehensive analysis using ImageJ software to extract key kinematic data. For micromachine actuation studies, specially fabricated samples were utilized. These samples were developed using SU‐8 photoresist developer and rinsed with isopropyl alcohol (IPA) to achieve clean, high‐fidelity structural patterns. The micro‐generator's actuation mechanism relied on a sophisticated approach combining transverse acoustic waves propagating through the glass substrate with a longitudinal wave traveling within the liquid medium. This dual‐wave excitation system proved essential for inducing and effectively tracking the micro‐generator's motion, which was meticulously recorded via the aforementioned optical microscopy setup.

In fluid transport characterization experiments, the micro‐generator was initially immersed in deionized (DI) water, resulting in the entrapment of microbubbles within its engineered surface cavities. Subsequently, a suspension of 10‐µm tracer particles was introduced into the DI water bath using a precision pipette (Dragonlab, range: 1–100 µL). Ultrasound excitation, with precisely tunable frequency and amplitude, was then applied to activate the micro‐generator. The resulting acoustic streaming propelled the tracer particles, enabling visualization and quantification of the induced fluid flow.

To analyze tracer particle motion under varying flow conditions, videos were recorded at 140 fps using an RH‐2000 microscope. Particle trajectories were accurately determined using the Manual Tracking plugin in ImageJ. Notably, the trajectories presented in Figures S2 and S5 represent partial particle paths; some particles moved out of the focal plane or appeared only intermittently during observation. Flow velocities reported in Figure 2c,d were derived by averaging the instantaneous velocities of individual particles, calculated from their initial detection within the monitored region until their complete exit from the delivery structure. This averaging methodology reliably characterizes the flow field generated by microgenerator actuation.

## Author Contributions

L.W. and T.‐Y.H. conceived the project. L.W. and Y.W. developed the fabrication process. L.W. and Y.T. developed the design strategy of reconfigurable acoustofluidic microgenerators. T.‐Y.H., L.W., H.W., X.Z. and T.L. worked on the manuscript together. All authors contributed to the discussion of the results and the manuscript revision.

## Conflicts of Interest

The authors declare no conflicts of interest.

## Supporting information




**Supporting File 1**: advs73659‐sup‐0001‐SuppMat.docx.

**Supporting File 2**: advs73659‐sup‐0002‐Movie S1‐S7.zip.

## Data Availability

The data that support the findings of this study are available from the corresponding author upon reasonable request.
