# Supplementary material for: Reconfigurable Acoustofluidic Microvortices for Selective Microcargo Delivery
Source: Adv Sci (Weinh). 2026 Jan 9;13(16):e21612. doi: 10.1002/advs.202521612 (PMC13042385; doi:10.1002/advs.202521612)
Supplement: Supplementary file 1 — Supporting File 1: advs73659‐sup‐0001‐SuppMat.docx. [file ADVS-13-e21612-s002.docx]

**Supporting Information for**

**Reconfigurable acoustofluidic microvortices for selective microcargo delivery**

*Lei Wang^1^, Yiqiang Wu^1^, Yiyang Tang^1^, Hui Wei^1^, Tianshi Lyu^2^, Xin Zhang^3^, Tian-Yun Huang^1,4,*^*

**Lei Wang, Yiqiang Wu, Yiyang Tang, Hui Wei, Tian-Yun Huang**

^1^Laboratory for Micromachine Intelligence, School of Advanced Manufacturing and Robotics, PKU Research Center for Robotics, Peking University, Beijing, 100871, China

**Tianshi Lyu**

^2^Department of interventional and vascular surgery, Peking University First Hospital, Beijing, 100034, China

**Xin Zhang**

^3^Department of Sports Medicine, Peking University Third Hospital, Institute of Sports Medicine of Peking University, Beijing, 100191, China.

**Tian-Yun Huang**

^4^National Key Laboratory of Advanced Micro and Nano Manufacture Technology, Beijing, 100871, China

E-mail: [huangtianyun@pku.edu.cn](mailto:huangtianyun@pku.edu.cn)

**This PDF file includes**

Supporting Text

Figure S1 to Figure S14

Supporting Information References

**Other Supporting Materials for this manuscript:**

Movies S1 to S7

**Supporting Text**

The setup of the two-microbubble interaction, shown in **Figure S8**, features L as the distance between the microbubbles’ equilibrium centers. In the ultrasonic manipulation environment examined, microbubbles are assumed to be in an unbounded liquid. The wavelength of the acoustic waves causing microbubble oscillations is much larger than both the microbubbles’ radius and their separation distance. Therefore, we treat the surrounding liquid as incompressible and ignore viscous effects. Additionally, given the amplitude of the acoustic waves generated by the applied voltage, nonlinear effects are neglected. We focus only on linear oscillations of the microbubbles under steady-state conditions where the excitation frequency equals their natural frequency. The primary Bjerknes force calculation proceeds as follows ^[1][2]^:

Assuming spherical microbubbles with dimensions significantly smaller than the ultrasonic excitation wavelength, the primary Bjerknes force acting on the microbubbles can be expressed in tensor form as follows:

$F_{i}=\int_{S} \sigma_{ik}n_{ik}dS$ (S1)

*S* is the surface area of microbubbles. *n* is the unit outward normal relative to *S*. $\sigma_{ik}$ is the stress tensor in the liquid. Then equation (1) can be expressed in vector form as:

$F=-\int_{S} pndS$ (S2)

*p* is the liquid pressure. Assuming that *p* has no singularities within the body. Equation (S2) can be transformed into:

$F=-\int_{V} \nabla pdV$ (S3)

Where *V* represents the volume of the microbubble. Assuming that the center of mass of the microbubble at time *t* is located at position *r*, since the microbubble is very small, the spatial variation of *∇p* within the volume of the microbubble can be ignored. Then equation (S3) can be expressed as:

$F=-\int_{V} \nabla pdV\approx-V\left( t \right)\nabla p\left( r,t \right)$ (S4)

This equation is used to calculate the primary Bjerknes force received by microbubbles under ultrasonic excitation. Considering that the primary Bjerknes force is an average force, equation (S4) can be rewritten as:

$F=-\frac{4\pi}{3}\left\langle R^{3}(t)\nabla p_{ac}(r,t) \right\rangle$ (S5)

Wherein, $\left\langle\right\rangle$ represents the time average, and *R(t)* is the pulsation of the microbubble radius over time. $p_{ac}=A(r)exp(-i\omega t)$ is the driving sound pressure, 𝐴(𝑟) is the amplitude, and 𝜔 is the angular frequency. If *𝑅_0_* is the radius of the microbubble when it is stationary and the excitation sound field is weak, the radial linear oscillation equation of the microbubble is:

$\ddot{x}\left( t \right)+\omega\delta\dot{x}\left( t \right)+\omega_{0}^{2}x\left( t \right)=-\frac{p_{ac}(r,t)}{\rho_{0}R_{0}}$ (S6)

Among them, $\rho_{0}$ is the density of the liquid medium, $\omega_{0}$ is the resonance angular frequency of the microbubble. $\delta=\delta_{rad}+\delta_{th}+\delta_{vis}$ is the total damping constant including radiation damping, thermal damping and viscous dissipation damping. The resonant frequency and damping constant are calculated by the following formula:

$\omega_{0}=\frac{1}{R_{0}}\left[ \frac{3\gamma_{ef}}{\rho_{0}}\left( p_{0}+\frac{2\sigma}{R_{0}} \right)-\frac{2\sigma}{\rho_{0}R_{0}} \right]^{1/2}$ (S7)

$\delta_{rad}=kR_{0}$ (S8)

$\delta_{th}=d_{th}\left( \frac{\omega_{0}}{\omega} \right)^{2}$ (S9)

$\delta_{vis}=\frac{4\eta}{\omega\rho_{0}R_{o}^{2}}$ (S10)

where

$\gamma_{ef}=\gamma\left[ \left( 1+d_{th}^{2} \right)\left( 1+\frac{3(\gamma-1)(sinhX-sinX)}{X(coshX-cosX)} \right) \right]^{1/2}$ (S11)

$d_{th}=3(\gamma-1)\left[ \frac{X\left( sinhX+sinX \right)-2\left( coshX-cosX \right)}{X^{2}\left( coshX-cosX \right)+3(\gamma-1)X(sinhX-sinX)} \right]$ (S12)

where $p_{0}$ is the net hydrostatic pressure in the liquid medium, $\sigma$ is the surface tension, $k=\frac{\omega}{c}$ is the wave number of the liquid. c is the sound velocity of the liquid medium, *η* is the shear viscosity of the liquid, and *γ* is the specific heat ratio of the gas in the microbubble. In equations (S11) and (S12), the quantity *X* is calculated as follows:

$X=R_{0}\sqrt{\frac{2\omega}{\chi_{g}}}$ (S13)

where $\chi_{g}$ is the thermal diffusivity of the gas in the microbubble, given by:

$\chi_{g}=\frac{\kappa_{g}}{\left( c_{pg}\rho_{g0} \right)}$ (S14)

$\kappa_{g}$ is the thermal conductivity of the microbubble gas, $c_{pg}$ is the specific heat of the gas at constant pressure. $\rho_{g0}$ is the equilibrium density of the gas, given by:

$\rho_{g0}=\rho_{gA}\frac{\left( p_{0}+\frac{2\sigma}{R_{0}} \right)}{p_{A}}$ (S15)

where $\rho_{gA}$ is the gas density at atmospheric pressure $p_{A}$.

According to equation (S6), we have:

$x\left( t \right)=-\frac{p_{ac}(r,t)}{\rho_{0}R_{0}(\omega^{2}-\omega_{0}^{2}+i\omega^{2}\delta)}$ (S16)

By substituting Equation (S16) into Equation (S5) through the time-averaged product of two complex quantities and retaining acoustic pressure amplitude terms up to second order, it can be demonstrated that

$F=\frac{2\pi R_{0}}{\rho_{0}}Re\left\{ \frac{A^{*}(r)\nabla A(r)}{\omega^{2}-\omega_{0}^{2}+i\omega^{2}\delta} \right\}$ (S17)

Under the excitation of a plane traveling wave, $A\left( r \right)=A_{m}exp(ik\cdot r)$. Under the excitation of a plane standing wave, $A\left( r \right)=A_{m}cos(k\cdot r)$. $A_{m}$ is the complex pressure amplitude and $k$ is the wave vector in the liquid.

The secondary Bjerknes force is calculated below^[2][3]^:

Since microbubbles are small and spherical, the secondary Bjerknes force between bubbles can be calculated by equation (S4). In equation (S4), assume that $V\left( t \right)=V_{1}(t)$ is the volume of the first microbubble changing with time, and is the liquid pressure generated by the scattered wave of the second microbubble. Then:

$F_{B}=-\left\langle V_{1}(t)\nabla p_{2}\left( r_{2},t \right) \right\rangle|_{r_{2}=L}$ (S18)

Under the linear approximation, the microbubble volume $V_{1}(t)$ can be written as:

$V_{1}\left( t \right)=\frac{4}{3}\pi R_{1}^{3}(t)=\frac{4}{3}\pi\left[ R_{10}+x_{1}(t) \right]^{3}\approx\frac{4}{3}\pi R_{10}^{3}\left[ 1+3\frac{x_{1}(t)}{R_{10}} \right]$ (S19)

where $R_{10}$ is the equilibrium radius of first microbubble, and $x_{1}(t)$ represents the linear variation of the radius of the microbubble. Approximately, the velocity potential generated by the radial oscillation of second microbubble is given by:

$\varphi_{2}\left( r_{2},t \right)=-\frac{R_{2}^{2}(t)\dot{R}_{2}(t)}{r_{2}}\approx\frac{R_{20}^{2}\dot{x}_{2}(t)}{r_{2}}$ (S20)

Substituting equation (S19) and equation (S21) into equation (S18), we obtain:

$F_{B}=-\frac{2\pi\rho_{0}R_{10}^{2}R_{20}^{2}}{L^{2}}Re\left\{ x_{1}^{*}(t)\ddot{x}_{2}(t) \right\}$ (S22)

$F_{B}$ represents the magnitude of the force including the sign. $F_{B}>0$ means that the microbubbles attract each other, and $F_{B}<0$ means that the microbubbles repel each other. Assuming that the change in the amplitude of the incident sound field pressure can be ignored at the microbubble scale level, the expression of the interaction force between microbubbles can be obtained as:

$F_{B}=\frac{2\pi R_{10}R_{20}\left| A_{m} \right|^{2}\left[ \left( 1-\frac{\omega_{1}^{2}}{\omega^{2}} \right)\left( 1-\frac{\omega_{2}^{2}}{\omega^{2}} \right)+\delta_{1}\delta_{2} \right]}{\rho{\omega^{2}L}^{2}\left[ \left( 1-\frac{\omega_{1}^{2}}{\omega^{2}} \right)^{2}+\delta_{1}^{2} \right]\left[ \left( 1-\frac{\omega_{2}^{2}}{\omega^{2}} \right)^{2}+\delta_{2}^{2} \right]}$ (S23)

Among them, $\omega_{1}$ and $\omega_{2}$ are the eigenfrequencies of microbubbles, and $\delta_{1}$ and $\delta_{2}$ are their total damping coefficients.

From equation (S23), we can see that when $\omega_{1},\omega_{2}<\omega$ or $\omega_{1},\omega_{2}>\omega$, the bubbles attract each other, and repulsion only occurs when $\omega$ is between $\omega_{1},\omega_{2}$.

**Supporting figures:**


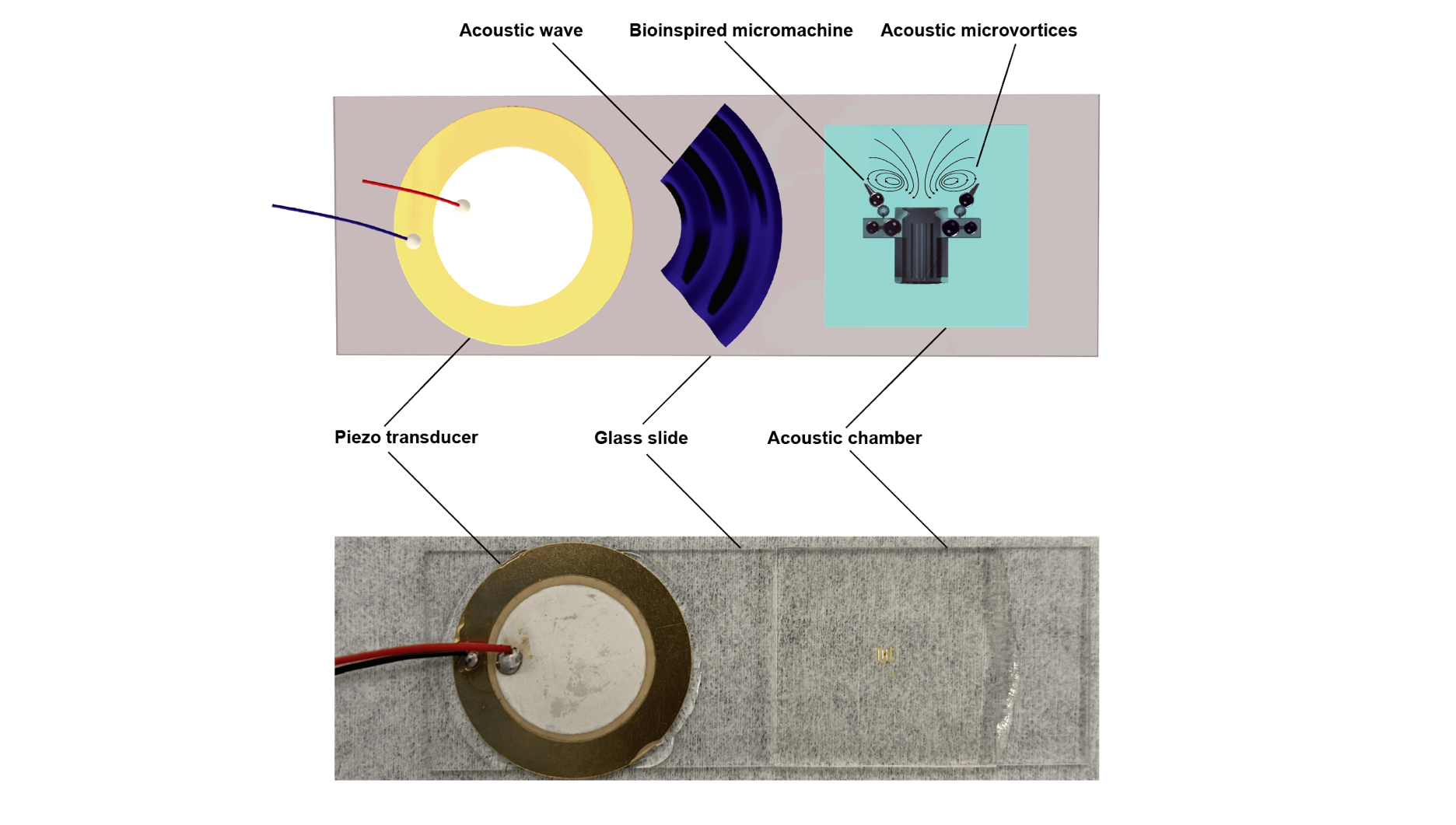


**Figure S1.** The acoustic system was constructed on a 25 mm by 75 mm by 1 mm glass slide, with a transducer disc attached using epoxy resin.


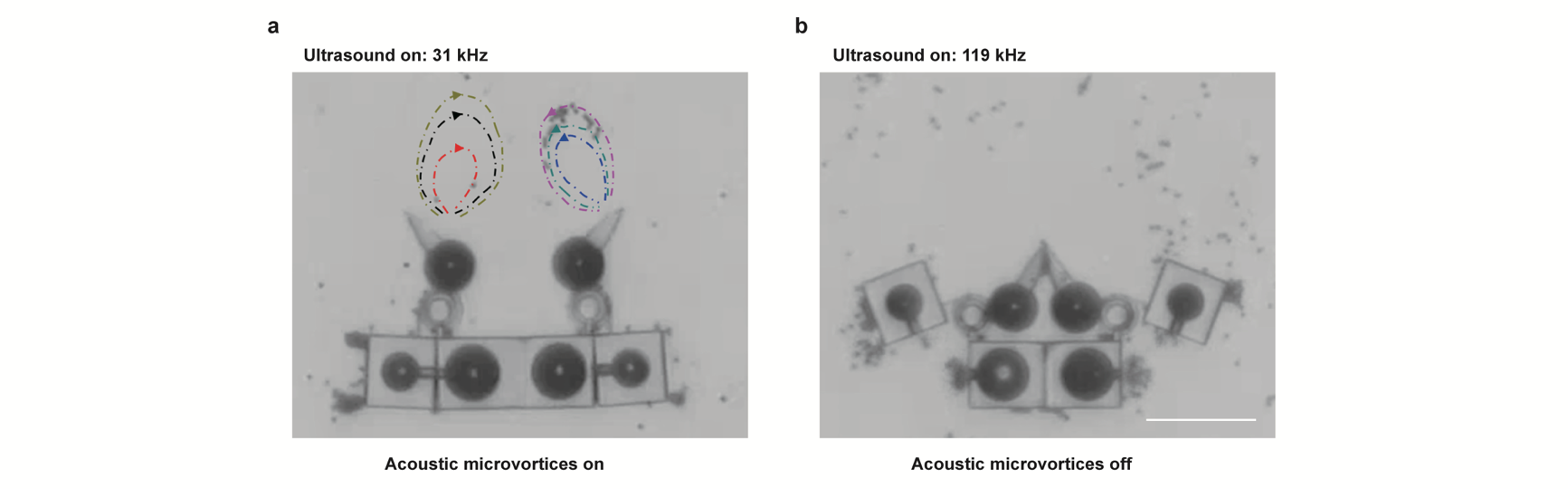


**Figure S2.** The microscope images of acoustic microvortex generator with the microvortex turned **a** on and **b** off. The dotted line indicates the path of the acoustic vortices. Scale bar, 100 μm.


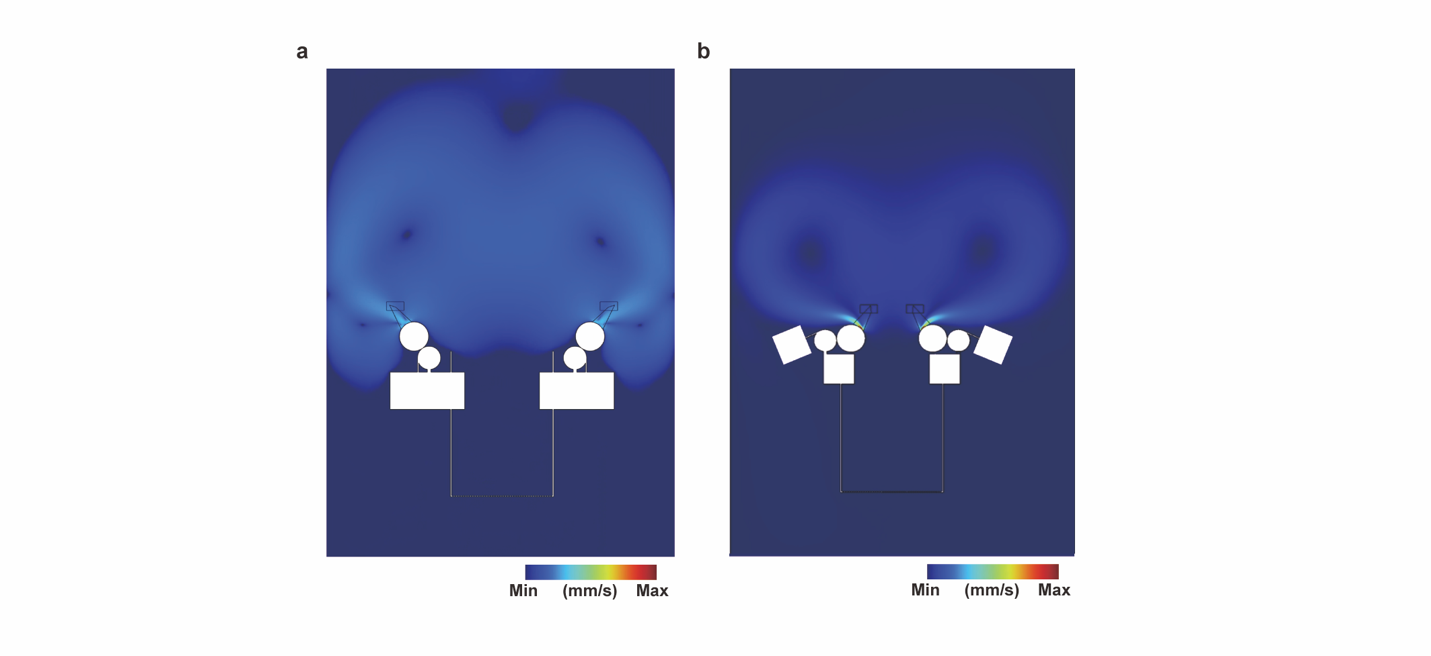


**Figure S3.** The numerical simulations of the microstreaming velocity in a compound micromachine: **a** the capture mode and **b** the release mode. The color bars indicate the normalized streaming velocities.


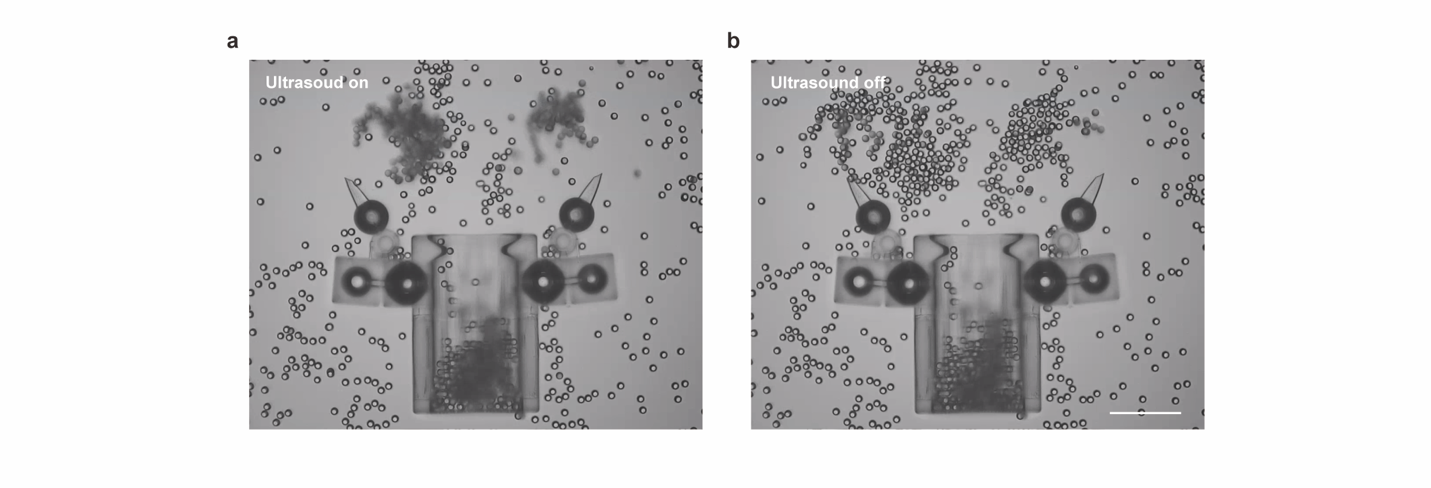


**Figure S4.** The capture process operates at low Reynolds numbers, starting and ending within milliseconds of activating or deactivating the input source. **a** The cargoes spin within microvortices during ultrasonic excitation. **b** When the ultrasonic stimulation ceases, the cargoes slowly diffuse away. Scale bar: 100 μm.


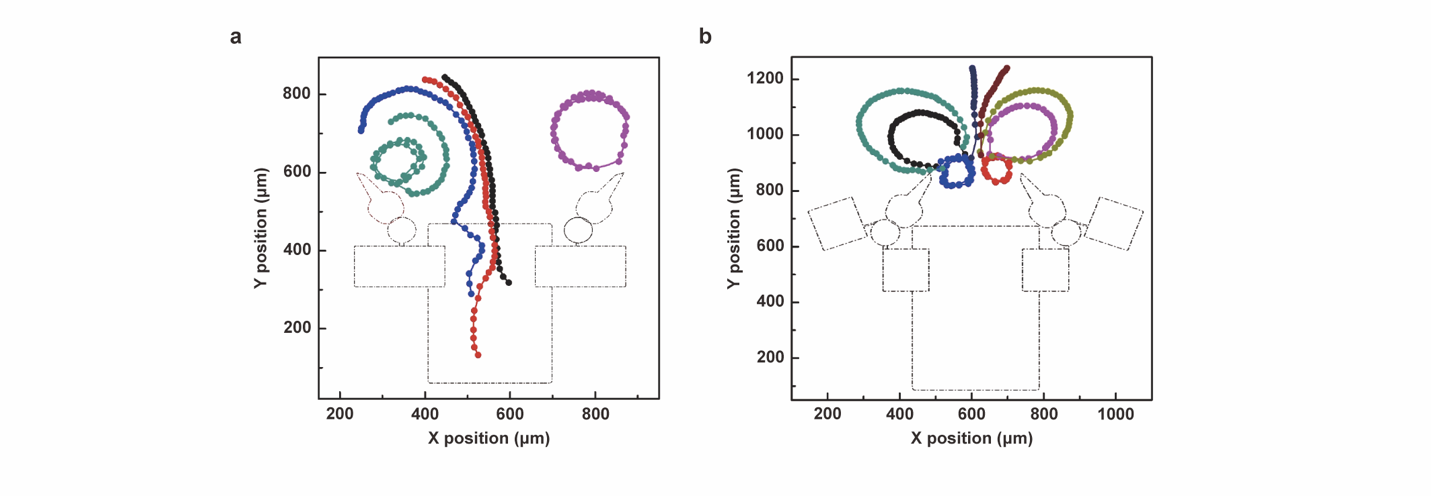


**Figure S5.** The spatial trajectories of cargoes during capture and release by the compound micromachine: **a** capture mode and **b** release mode.


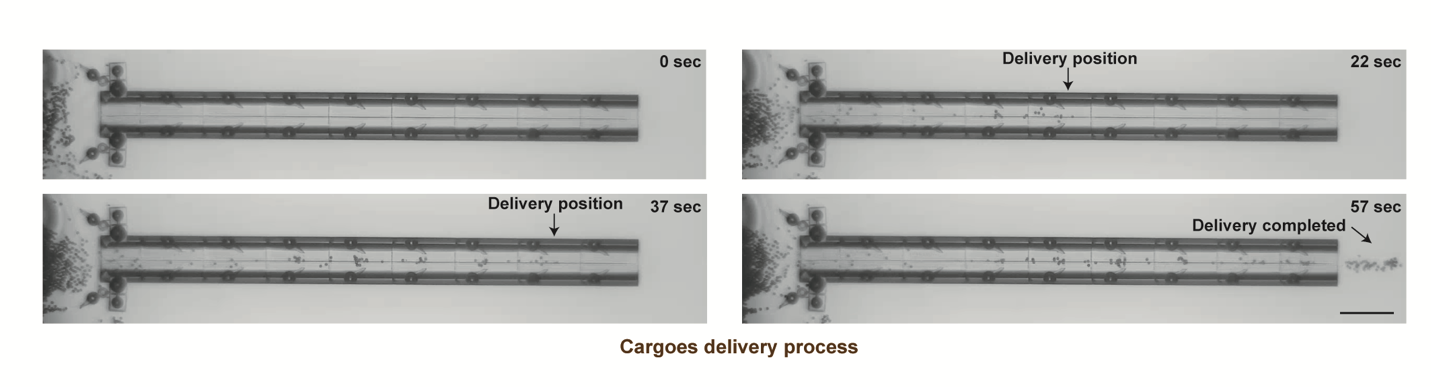


**Figure S6.** Image sequence demonstrating cargoes becoming captured at 31 kHz and 10 VPP. Scale bar, 200 μm.


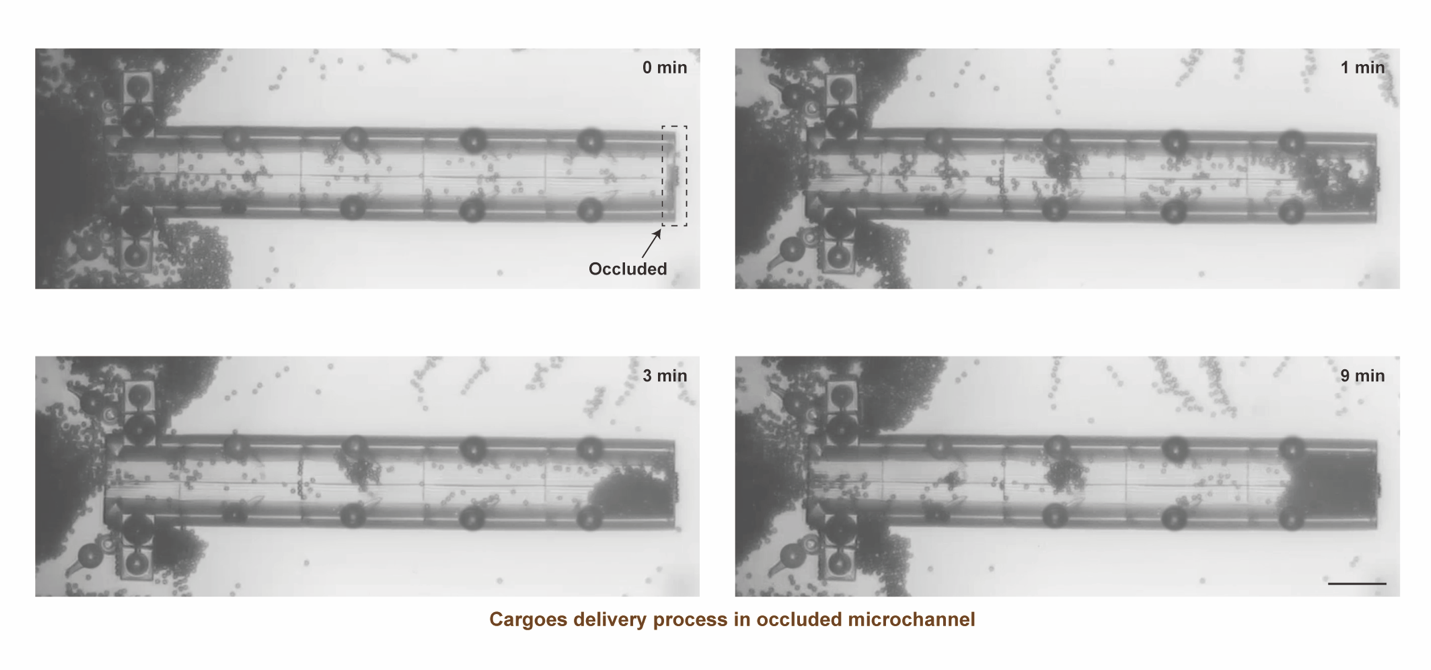


**Figure S7.** Microscope images of the cargo delivery process in the occluded channel. Image sequence demonstrating cargoes becoming captured at 31 kHz and 10 VPP. Scale bar, 100 μm.


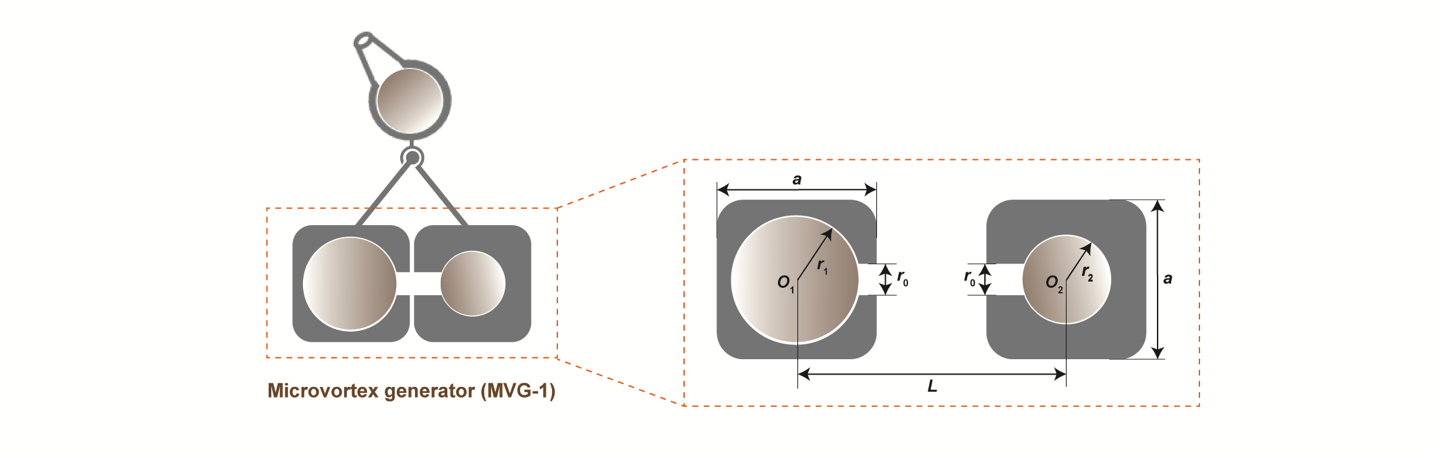


**Figure S8.** A schematic diagram of the structural dimensions of the microvortex generator with microbubbles immersed in the liquid medium.


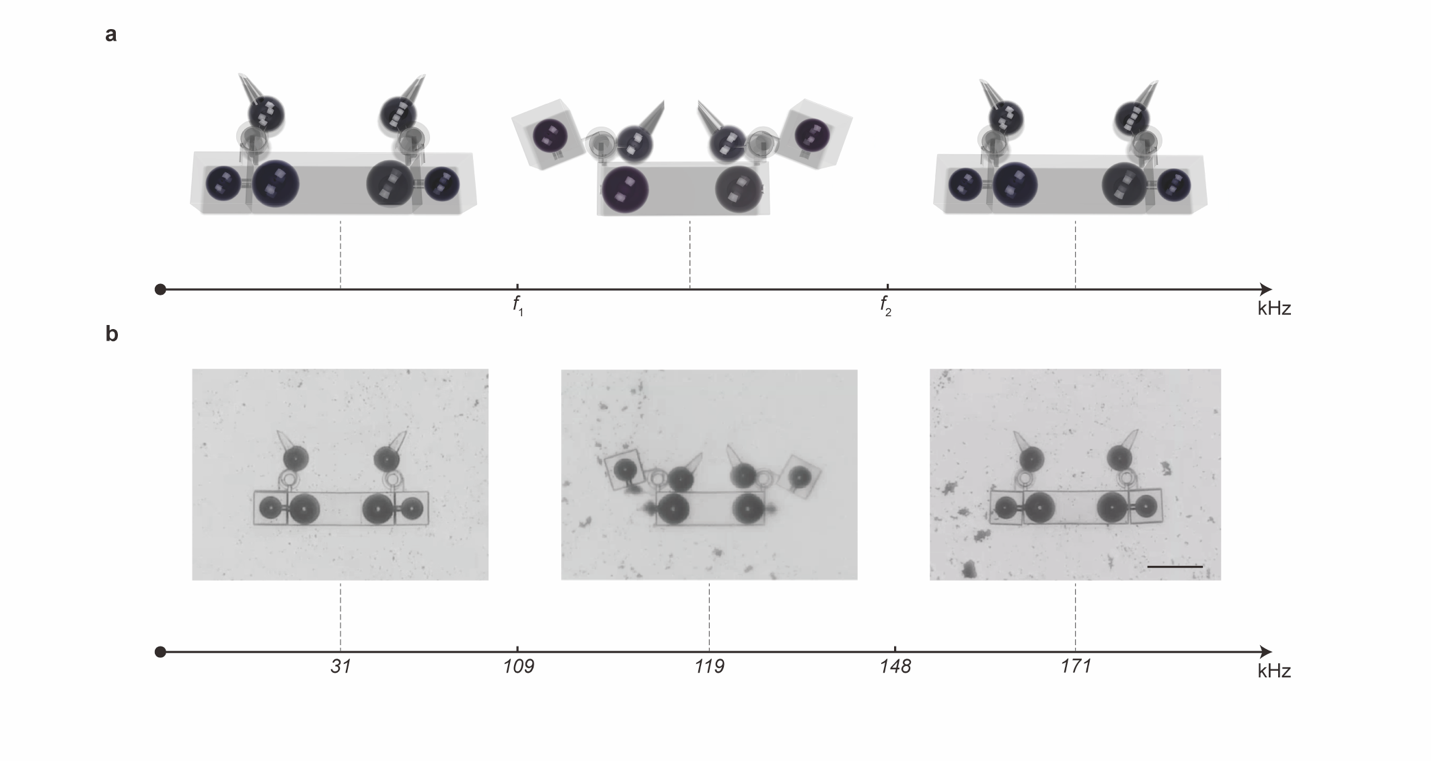


**Figure S9.** displays acoustic bandwidth division with r-MVGs. **a** The locations of r-MVGs in the rotifer-inspired micromachine across the ultrasound frequency band are shown, with insets illustrating the corresponding modes. **b** Microscope images depict microbubbles moving away from or attracted to each other under varying ultrasound excitation frequencies. Scale bar, 100 μm.


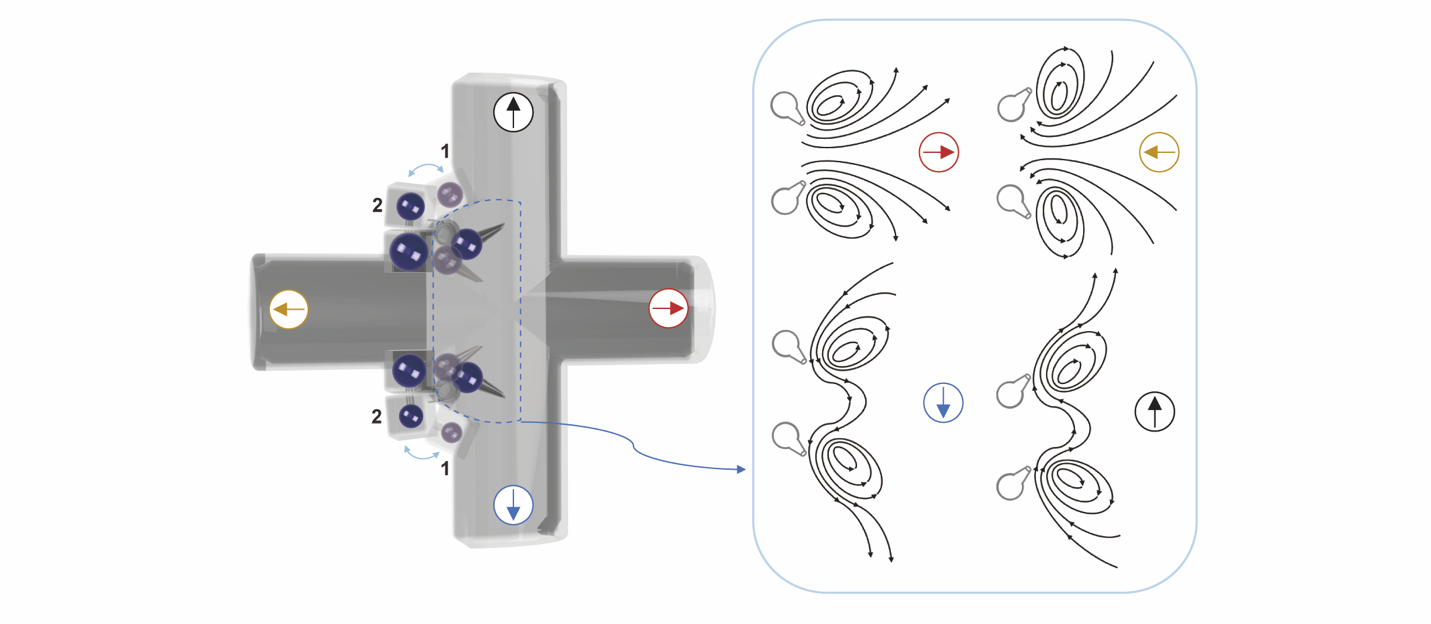
 **Figure S10.** Positional relationship and flow field distribution of the r-MVG-2 at the intersection of the channels under different modes.


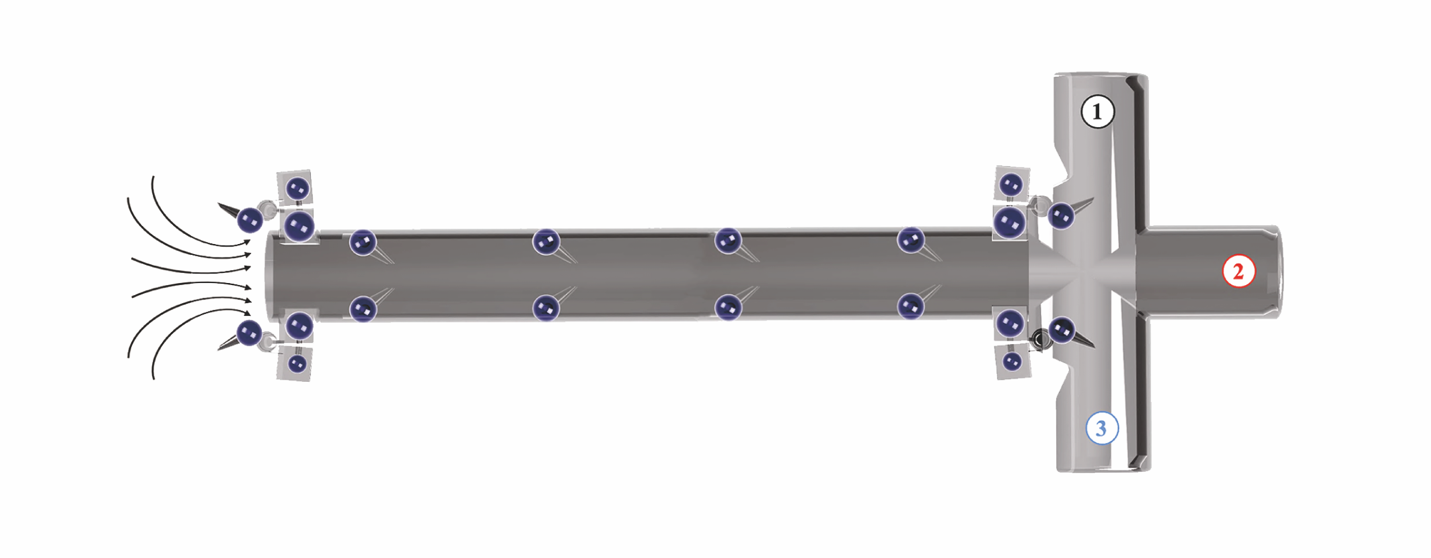


**Figure S11.** Schematic of selective delivery channel network with embedded microbubbles. Arrows of different colors indicate that the net flow direction of the channel can be selected separately. Microbubbles embedded in the channels increase the stability of the delivery device.


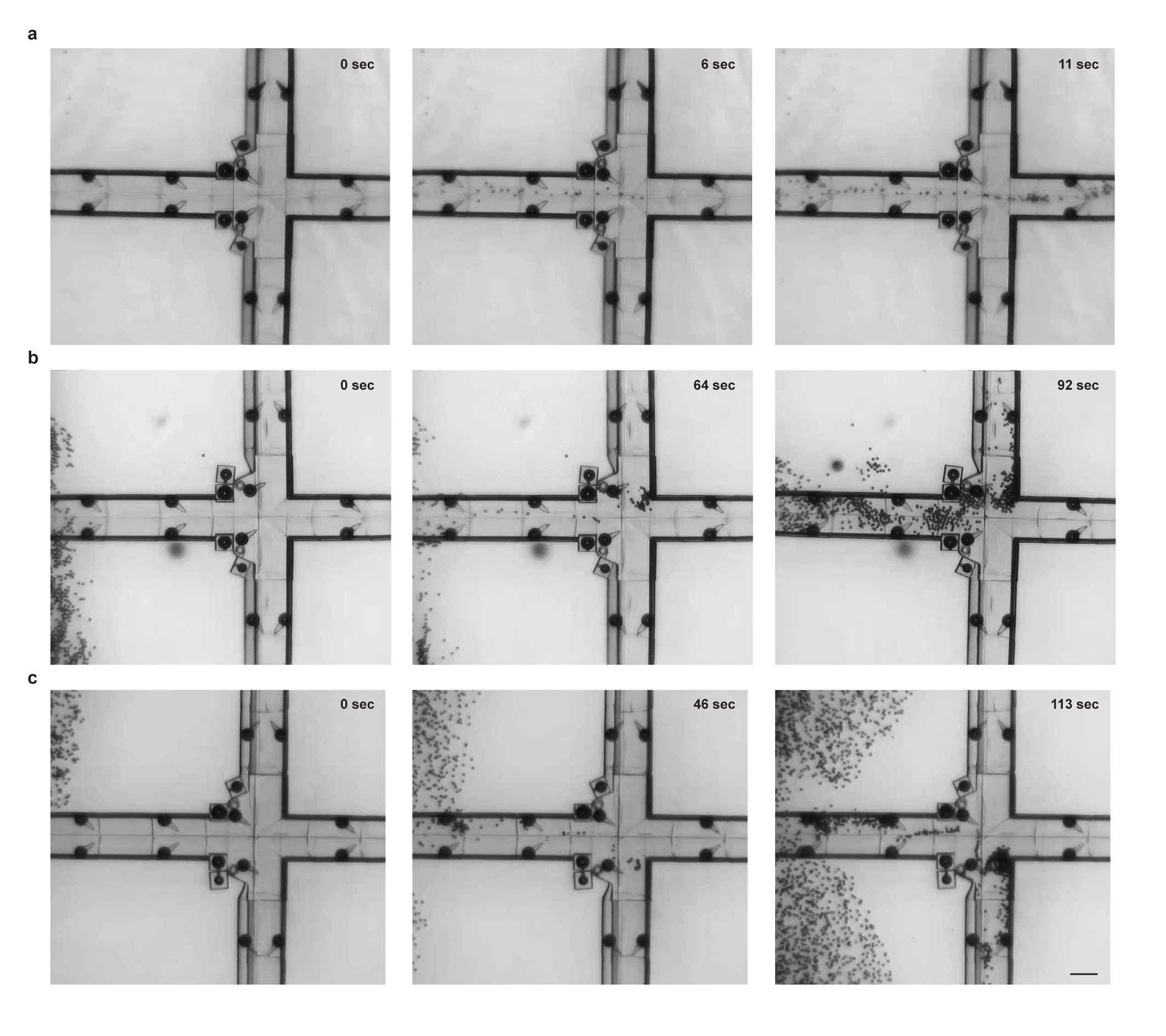


**Figure S12.** Selective cargo delivery in the selective delivery channel network with embedded microbubbles. **a-c** The microscope images show the aggregation and collective motion of cargoes at different time steps. Under continuous excitation at 20 VPP, cargoes were delivered to the target channels. The blue arrow outside the channels indicates the movement position of the tracked cargoes in the channel. Scale bar, 100 μm.


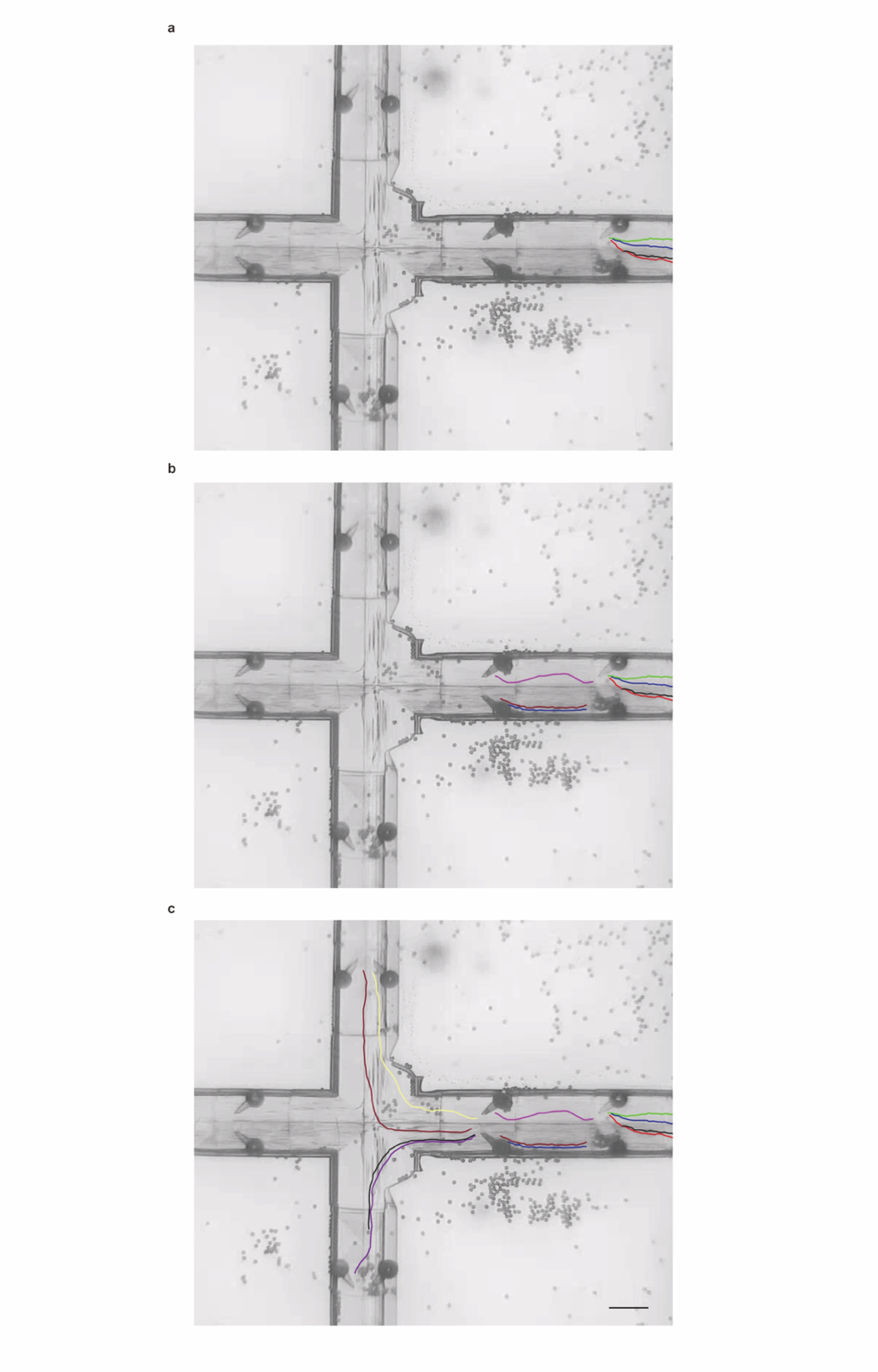


**Figure S13.** Control experiments without r-MVG-2 at microchannel intersections. **a-c** Microscope image of cargoes randomly entering channels without r-MVG-2. Scale bar, 100 μm.


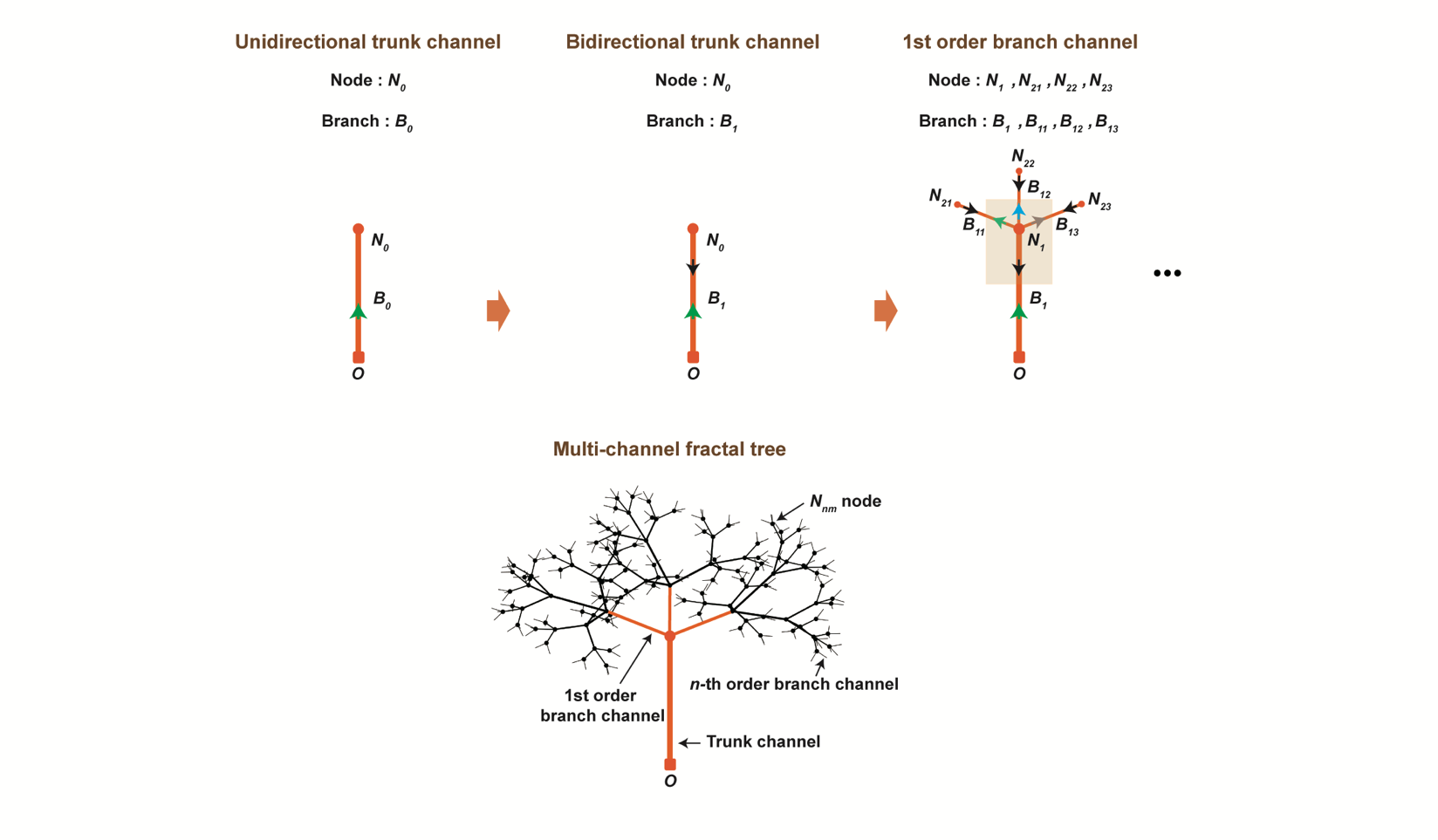


**Figure S14.** Multi-channel fractal tree designs and experimental verification. The evolution process of multi-channel fractal tree.


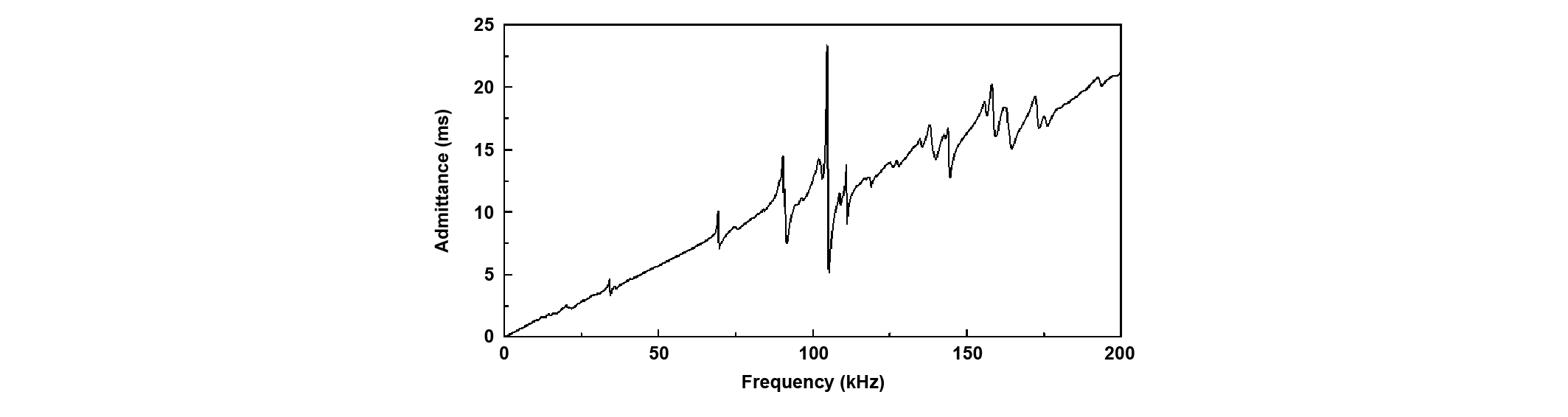


**Figure S15.** Admittance curve of the transducer.


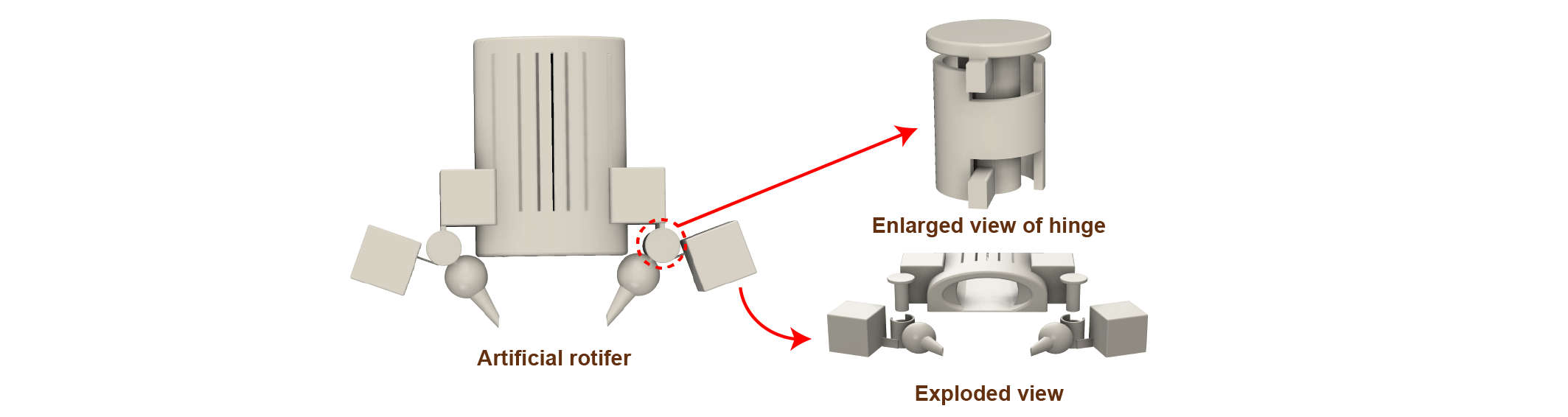


**Figure S16.** Exploded view of the artificial rotifer and an enlarged view of hinge.


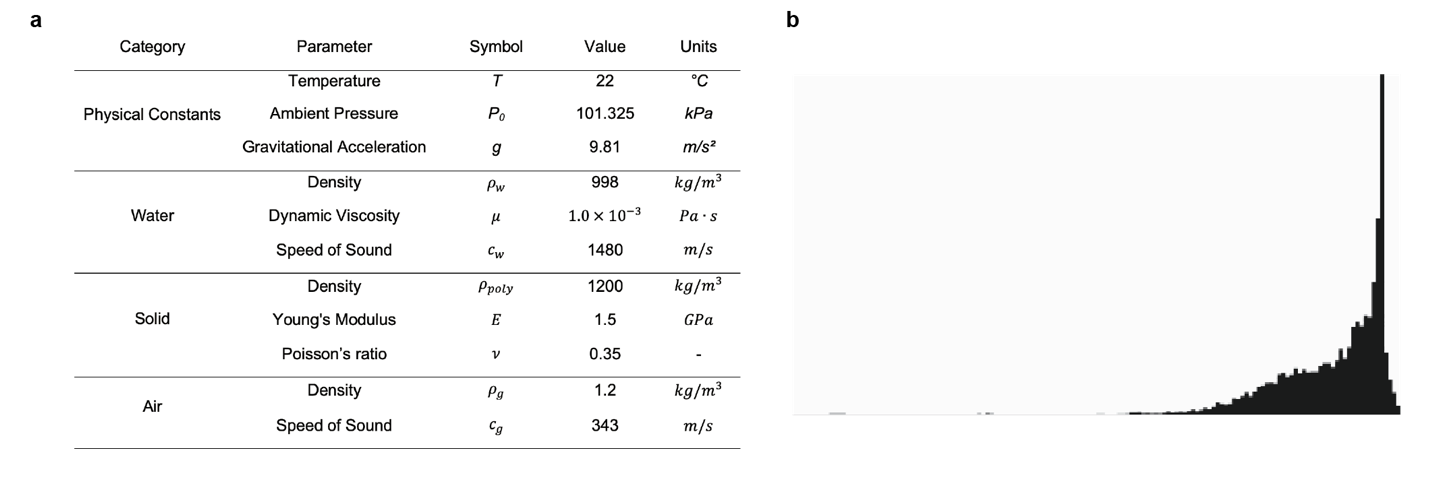


**Figure S17.** Simulation parameter settings **a** and mesh quality distribution **b** in COMSOL.


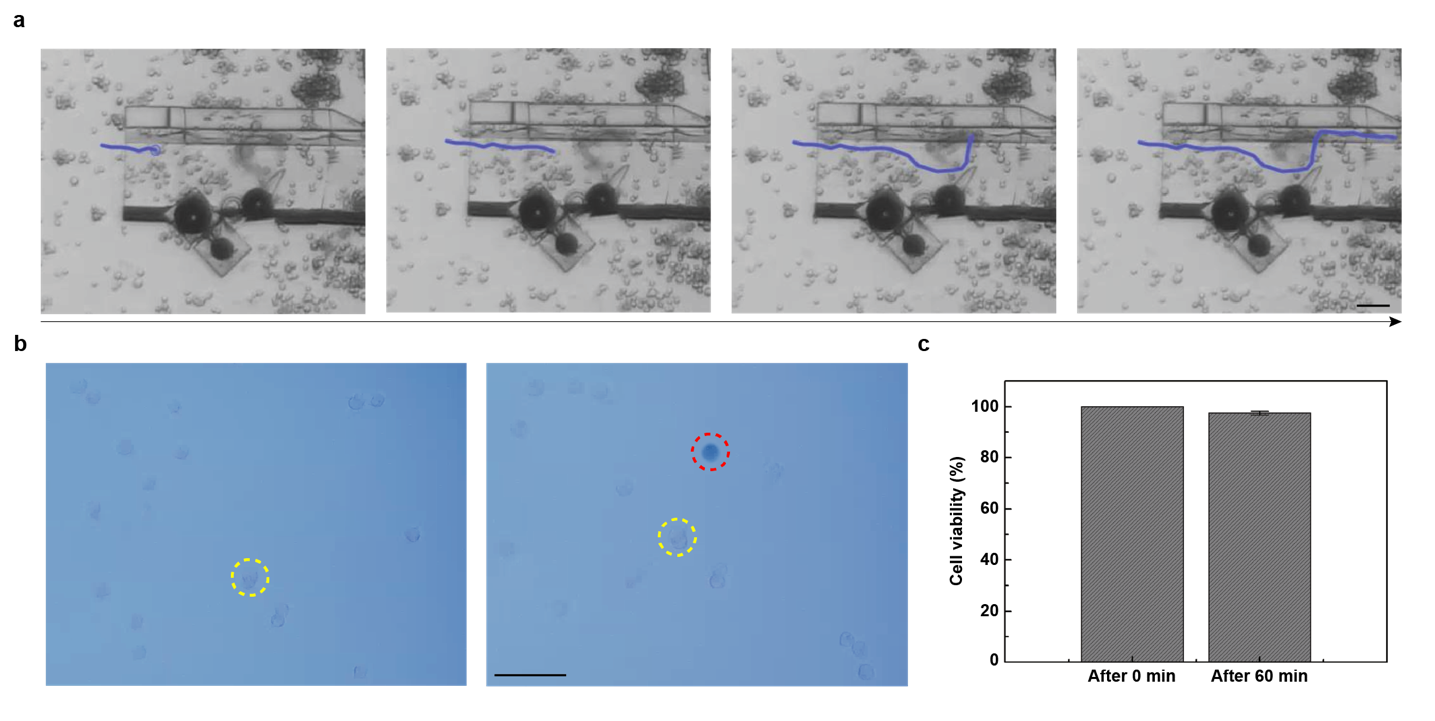


**Figure S18.** Compatibility of delivery devices with biological payloads under 30 Vpp ultrasonic excitation. (a) Image sequence showing the delivery of cells. (b) . After acoustic excitation, most drosophila cells were still alive. The cells highlighted in a yellow circle demonstrates a living cell; the cell marked by a red circle indicates a dead one. The drosophila cell viability was evaluated at 0 min and 60 min; (c) cell viability test results before and after 60 min acoustic delivery.

**Supporting Information References:**

1. Doinikov A A, Zavtrak S T, *Physics of Fluids*, 1995, 7(8): 1923-1930.
2. Doinikov A A, *Bubble and particle dynamics in acoustic fields: modern trends and applications*, 2005, 661: 95-143.
3. Pandey, Vikash, *Physical Review E* 2019, 99(4): 042209.
